# Livestock-associated risk factors for pneumonia in an area of intensive animal farming in the Netherlands

**DOI:** 10.1371/journal.pone.0174796

**Published:** 2017-03-31

**Authors:** Gudrun S. Freidl, Ineke T. Spruijt, Floor Borlée, Lidwien A. M. Smit, Arianne B. van Gageldonk-Lafeber, Dick J. J. Heederik, Joris Yzermans, Christel E. van Dijk, Catharina B. M. Maassen, Wim van der Hoek

**Affiliations:** 1 Centre for Infectious Diseases, Epidemiology and Surveillance, Centre for Infectious Disease Control, National Institute for Public Health and the Environment (RIVM), Bilthoven, the Netherlands; 2 European Programme for Intervention Epidemiology Training (EPIET), European Centre for Disease Prevention and Control (ECDC), Stockholm, Sweden; 3 Institute for Risk Assessment Sciences (IRAS), Division of Environmental Epidemiology, Utrecht University, Utrecht, the Netherlands; 4 Netherlands Institute for Health Services Research (NIVEL), Utrecht, Netherlands; Universite de Bretagne Occidentale, FRANCE

## Abstract

Previous research conducted in 2009 found a significant positive association between pneumonia in humans and living close to goat and poultry farms. However, as this result might have been affected by a large goat-related Q fever epidemic, the aim of the current study was to re-evaluate this association, now that the Q-fever epidemic had ended. In 2014/15, 2,494 adults (aged 20–72 years) living in a livestock-dense area in the Netherlands participated in a medical examination and completed a questionnaire on respiratory health, lifestyle and other items. We retrieved additional information for 2,426/2,494 (97%) participants from electronic medical records (EMR) from general practitioners. The outcome was self-reported, physician-diagnosed pneumonia or pneumonia recorded in the EMR in the previous three years. Livestock license data was used to determine exposure to livestock. We quantified associations between livestock exposures and pneumonia using odds ratios adjusted for participant characteristics and comorbidities (aOR). The three-year cumulative frequency of pneumonia was 186/2,426 (7.7%). Residents within 2,000m of a farm with at least 50 goats had an increased risk of pneumonia, which increased the closer they lived to the farm (2,000m aOR 1.9, 95% CI 1.4–2.6; 500m aOR 4.4, 95% CI 2.0–9.8). We found no significant associations between exposure to other farm animals and pneumonia. However, when conducting sensitivity analyses using pneumonia outcome based on EMR only, we found a weak but statistically significant association with presence of a poultry farm within 1,000m (aOR: 1.7, 95% CI 1.1–2.7). Living close to goat and poultry farms still constitute risk factors for pneumonia. Individuals with pneumonia were not more often seropositive for *Coxiella burnetii*, indicating that results are not explained by Q fever. We strongly recommend identification of pneumonia causes by the use of molecular diagnostics and investigating the role of non-infectious agents such as particulate matter or endotoxins.

## Introduction

In the Netherlands, the number of intensive livestock farms doubled within the first decade of the 21^st^ century [1]. Although the total number of farms has decreased over the past decades, the number of farm animals has increased [2]. This trend has raised concern about potential negative health effects on residents living close to intensive livestock farms. The debate between civil groups opposed to intensive livestock farming, the farming sector and policy makers was further fueled by the recent Q fever epidemic that occurred between 2007 and 2009 in the southern part of the Netherlands [3]. Caused by the zoonotic bacterium *Coxiella burnetii*, this epidemic resulted in more than 4000 notified human cases, mostly presenting as pneumonia [4]. Aborting dairy goats and dairy sheep were found to be the main cause of infection in humans, who were infected through inhalation of contaminated dust or aerosols distributed via the airborne route. Human cases started to decrease in 2010 in the Netherlands, coinciding with the introduction of veterinary interventions comprising of culling of pregnant goats and sheep on Q fever positive farms and vaccination of dairy goats and dairy sheep [3, 5]. Since the start of this vaccination campaign in early 2009, farms with at least 50 sheep or goats are obliged to vaccinate, which is strictly reinforced [6].

The health risks for residents living in the vicinity of livestock farms in the Netherlands were first addressed within the “Intensive Animal Husbandry and Health” study [7]. Within this project Smit et al. [8] studied the relationship between living in the vicinity of livestock-farms and Q fever or pneumonia diagnoses in 70,142 adults. Outcomes were retrieved from electronic medical records from general practitioners (GP) located in an area with Q fever positive farms during 2009. This study found that a high number of goats within 5km of the home address [quartile (Q) 4: 17,191–20,969 versus Q1: 0–2,250 goats] was indeed associated with Q fever [Adjusted Odds ratio (aOR) 12.03, 95% confidence interval (CI) 8.79–16.46] and pneumonia (aOR 1.86, 95% CI 1.28–2.21). Presence of poultry farms within 1km of the home address was also identified as a risk factor for pneumonia among adults (OR 1.25, 95% CI 1.06–1.47), which was hypothesized to be potentially linked to exposure to pathogens or air pollutants. Potential exposure to other pathogens, such as influenza viruses, and higher susceptibility to community-acquired pneumonia due to exposure to high levels of fine dust and endotoxins emitted by poultry farms were considered likely explanations of this finding [8, 9]. Residual confounding by goat exposure could not be fully ruled out in explaining the high incidence of pneumonia close to poultry farms.

Therefore, the aim of the current study was to re-investigate previously found associations between pneumonia and goat/ poultry exposure (as well as other types of animals) in the same study area in a period in which the Q-fever incidence among humans has dropped to pre-epidemic levels.

## Methods

### Study population and study design

As part of the “Livestock Farming and Neighbouring Residents’ Health” (VGO) project, a population-based cross-sectional study was conducted to investigate the relationship between adverse health effects in humans living close to livestock farms. A detailed description of the study design can be found in a project report [10].

In short, a questionnaire study was conducted among 14,882 adults living in the east of North-Brabant and the north of Limburg, an area characterized by a high density of livestock farms [11].

In total, 9220 (62%) gave consent to be contacted for further studies, of which 7180 (72%) met the following inclusion criteria and were invited to participate: (i) not working or living on a farm, and (ii) living within a 10 km radius of one of the 12 temporary research centers. Of these, 2494 (34.7%) participated in the medical examination which was conducted between March 2014 and February 2015 and included, among others, filling in an extended questionnaire comprising questions on demographics, respiratory health and lifestyle and providing a serum sample. We also investigated a possible association between having children in the household and pneumonia, as was shown in previous research [12, 13].

Electronic medical record (EMR) data were used, if (i) GPs registered according to certain quality criteria [8, 11] and (ii) if participants granted access to their EMR. EMR data were available through the NIVEL Primary Care Database of the Netherlands Institute for Health Sciences Research. Of the 2,494 participants, we excluded 68 individuals from the analysis. Of these, 66 did not provide consent to access their EMR data, whereas for two other individuals EMR data were unavailable. The final study population amounted to 2,426 individuals.

### Ethical aspects

The VGO study protocol was approved by the Medical Ethical Committee of the University Medical Centre Utrecht (protocol number 13/533). All 2,494 subjects signed informed consent. Patients’ privacy was ensured by keeping medical information and address records separated at all times by using a Trusted Third Party.

### Data availability statement

In consultation with the Medical Ethical Committee that approved the study protocol, data from the VGO study are not publicly available due to privacy protection of participants. The study’s privacy regulations stated that only researchers from NIVEL, IRAS, and RIVM (consortium partners) have access to the study database. Sharing an anonymized and de-identified dataset is not possible as it would still contain Electronical Medical Records and the personal data of participants, which could potentially lead to the identification of subjects. Researchers may reach a privacy agreement to access the data by contacting Prof. Dr. Dick Heederik (d.heederik@uu.nl) or Dr. L.A.M. Smit (l.a.smit@uu.nl).

### Data collection

#### Questionnaire

Study participants provided information on personal characteristics and lifestyle factors through the questionnaire, including age, gender, smoking habits, and education level. Body mass index (BMI) was calculated based on weight and height measured during the medical examination. In addition, information on respiratory diseases (i.e. pneumonia, COPD and asthma) and related risk factors and determinants, e.g. growing up on a farm or in the study area, keeping goats or poultry as a hobby, or reception of yearly influenza vaccination was collected.

#### Pneumonia outcome

**Fig 1:**
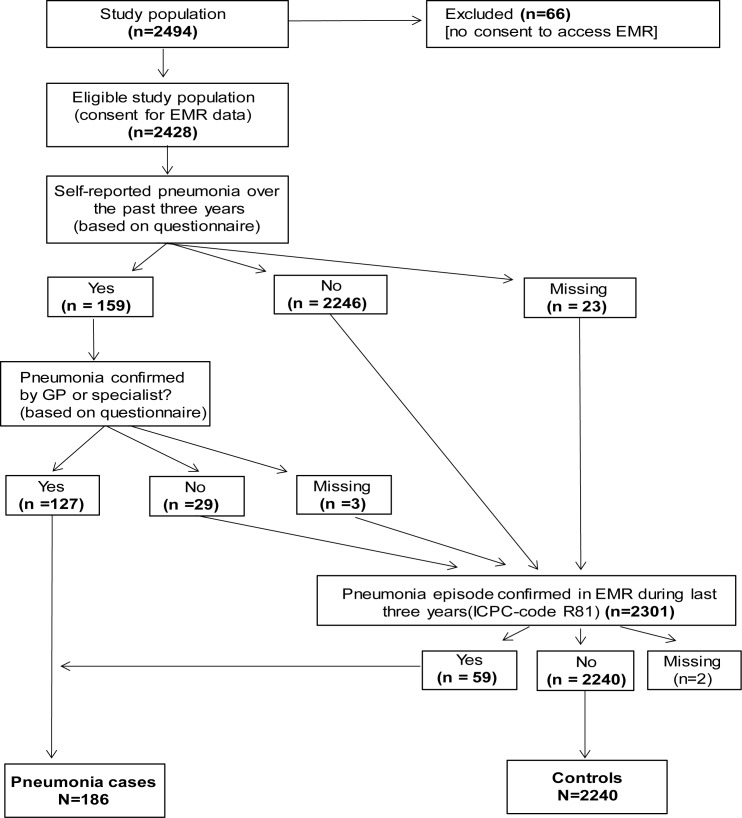
Construction of outcome variable ‘Having had pneumonia in the past three years’ based on information from questionnaires and electronic medical records (EMR).

‘Having had pneumonia in the three years preceding the medical examination’ (i.e. between 2012 and 2014/15) was defined as the outcome variable. We defined the outcome using two sources of information: (i) self-reported, physician-diagnosed pneumonia over the past three years as reported in the questionnaire, or (ii) having had at least one pneumonia episode recorded in the electronic medical record (EMR) during the three years preceding the medical examination (Fig 1). The benefit of combining data sources in order not to miss cases was described previously for COPD and asthma [14]. By asking for physician-diagnosed pneumonia in case of self-reporting we aimed to avoid misclassification bias. Information on pneumonia episodes was extracted from EMR using International Classification of Primary Care (ICPC) code ‘R81’. To account for possible recall bias of participants, we also conducted sensitivity analysis using ‘Having had a pneumonia episode registered in the EMR in the three years preceding the medical examination’ as an outcome.

#### Risk factors extracted from electronic medical records

We identified co-morbidities regarded as risk factor for pneumonia from the literature. Information on comorbidities of participants was similarly extracted from EMRs using ICPC codes. Individual risk factors were grouped into the following categories: chronic lung disease, chronic cardiovascular disease, cerebrovascular disease, chronic liver disease, chronic nephropathy, malignancies, auto-immune disease and neurological comorbidities (S2 Table).

#### Exposure to livestock farms

We obtained livestock data from provincial databases of mandatory environmental licenses for keeping livestock for 2012 to determine exposure to livestock [11]. Using a Geographic Information System (ArcGis), exposure to farms was quantified for each study participant individually based on geocodes of home address and farms (centroid of stable complex), as described previously [8]. The following variables were analyzed: (i) presence of livestock farms with a minimum number of animals (in increments of 500m) around the residence, (ii) distance between residence and poultry and goat farms expressed in quartiles, (iii) number of farms expressed in tertiles and (iv) number of animals expressed in tertiles (cattle, goats, horses, pigs, poultry, sheep) within 1000m of the home. As the distribution of number of goats within 1000m of their home address was highly skewed (S1 Fig), the construction of tertiles for this variable was not possible. Therefore, we decided to set the minimum number of goats to 50 in line with the threshold applied during the compulsory vaccination campaign during the Q-fever epidemic [6]. Farms were considered ‘Q-fever positive’ if they had experienced Q-fever-induced abortion waves or if they tested positive in bulk tank milk monitoring (data from GD Animal Health and the Food and Consumer Product Safety Authority).

#### Serology for Q fever

Serological analyses were performed using a commercial enzyme-linked immunosorbent assay (Serion ELISA classic, Virion/Serion, Würzburg, Germany) to test for IgG to C. burnetii phase II antigen. A titer of <20 IU/ml was considered negative, between 20 to 30 IU/ml borderline, and >30 IU/ml was classified seropositive. More details on the serological findings are described elsewhere [10].

#### Non-response analyses

To assess whether self-selection was present in this study, we conducted non-response analyses on different subpopulations. We first compared characteristics of individuals who were invited and responded (responders) or did not respond to the short questionnaire (non-responders), as well as individuals who were invited for the medical examination and participated (responders) or did not participate (non-responders).

### Statistical analysis

We used univariate logistic regression analysis (Wald Chi Square test statistic) to explore associations between risk factors related to lifestyle and livestock exposure and the occurrence of pneumonia, respectively. To adjust p-values retrieved from the univariate analyses for multiple testing, we used the Benjamini-Hochberg correction with a false discovery rate set to 10% [15]. We decided *a priori* to include age and gender in the multiple logistic regression models. Additional determinants with a p-value of less than 0.15 were also included in multivariable analyses investigating associations between livestock exposure and pneumonia. To retrieve the most accurate estimates for the odds ratio, multiple logistic regression models were used with three incremental sets of risk factors included in addition to livestock exposure variables: model A (age and gender), model B (age, gender, smoking, education level and BMI) and model C (age, gender, smoking, education level, BMI, chronic lung diseases and other comorbidities). Univariate and multiple logistic regression analyses were conducted in SAS 9.4 (SAS Institute Inc., Cary, NC, USA). For the multiple logistic regression models, co-morbidities described above–with exception of chronic lung disease–were combined in one dichotomous variable expressed as ‘Having had at least one registered episode of any listed comorbidity in the three years preceding the medical examination’. As ORs retrieved from the three adjusted models did not substantially differ in magnitude, this article focuses on results of the most parsimonious model, model A. Results of model B and C are shown in S3 Table. To rule out that associations between goat exposure and pneumonia were not due to Q fever, we analyzed associations between seropositivity against *C*. *burnetii* and pneumonia by creating a dichotomous variable in which seropositive and borderline results were grouped together.

To assess how many cases would be avoided if exposures were to be removed we used the following formula to calculate the population attributable fraction (PAF):

PAF = P_e_*[(adjusted OR -1)/ adjusted OR], where P_e_ is the proportion of cases that is exposed.

## Results

### Characteristics of study population

Among the 2,426 eligible study participants, we identified 186 pneumonia cases and 2,240 individuals without a history of recent pneumonia (Fig 1). Among the twelve research centers where medical examinations were conducted, location Heeswijk-Dinther had the highest percentage of pneumonia cases (16.3%), whereas the percentage was lowest in location Horn (3.7%, Table 1).

**Table 1:** Three-year cumulative frequency of pneumonia cases (defined as having had at least one pneumonia episode during the three years preceding the medical examination) including 95% confidence intervals (CI), overall and per location of the research center where the medical examination took place.

Research center	Total number of participants	Number of pneumonia cases	Percentage (95% CI)
**Heeswijk-Dinther**	369	60	16.3 (14.8–17.8)
**Heusden**	72	7	9.7 (8.5–10.9)
**Deurne**	129	11	8.5 (7.4–9.6)
**Afferden**	48	4	8.3 (7.2–9.4)
**Boxtel**	165	11	6.7 (5.7–7.7)
**Asten**	289	19	6.6 (5.6–7.6)
**Stramproy**	227	15	6.6 (5.6–7.6)
**Bakel**	305	18	5.9 (5.0–6.8)
**Someren**	169	10	5.9 (5.0–6.8)
**Budel**	186	10	5.4 (4.5–6.3)
**St. Anthonis**	386	18	4.7 (3.9–5.5)
**Horn**	81	3	3.7 (3.0–4.5)
**Total**	2,426	186	7.7 (6.6–8.8)

Study participants were between 20 and 72 years old. Pneumonia cases were slightly older than non-cases, with a median age of 61.8 [interquartile range (IQR) 54.9–66.9] versus 58.6 years (IQR 48.7–65.4), respectively (p-value<0.001). A comparison of other characteristics between cases and non-cases is shown in Table 2. In addition to gender, age and smoking, a low educational level, being under- and overweight, having chronic lung diseases and having other comorbidities were identified as risk factors (Table 2). Compared to non-cases (30.9%), pneumonia cases were more often vaccinated against influenza (46.2%) (Table 2). Influenza vaccination was strongly correlated with increasing age (Chi squared test, p-value <0.001) and was therefore excluded from multivariable analyses to avoid multicollinearity. Having grown up on a farm or outside the study area, or keeping goats or poultry for a hobby over the past five years did not constitute a risk factor (Table 2). Neither the presence, nor the number of children in the household (age categories: <4, 4–17, >17 years) were associated with pneumonia (data not shown). Adding ‘Living within 2000m of a Q-fever positive farm’ to the model did not change the OR between presence of goat farm within 500 to 2000m meters and pneumonia substantially and was therefore omitted from the model [range: OR 4.4, 95% CI 2.0–9.9 (within 500m) to OR 2.0, 95% CI 1.4–2.8 (within 2000m)].

**Table 2:** Characteristics of the study population (n = 2,426) and risk factors for pneumonia (186 pneumonia cases and 2,240 non-cases) based on univariate logistic regression analyses. Significant associations are depicted in bold face.

Characteristic		Pneumonia cases	Non-cases	Crude odds ratio	p-value^a^
		n (%)	n (%)	(95% CI)	
**Age categories**	≤49	34 (18.3)	573 (25.6)	Ref	0.004 ^d^
	>49 & ≤59	36 (19.4)	588 (26.3)	1.0 (0.6–1.7)	
	>59 & ≤66	61 (32.8)	571 (25.5)	**1.8 (1.2–2.8**)	
	>66	55 (29.6)	508 (22.7)	**1.8 (1.2–2.8**)	
**Gender**	Male	74 (39.8)	1038 (46.3)	Ref	0.086 ^e^
	Female	112 (60.2)	1202 (53.7)	1.3 (1.0–1.8)	
**Smoking habits**	Never smoked	65 (35.0)	959 (42.8)	Ref	0.114 ^e^
	Ex-smoker	101 (54.3)	1076 (48.0)	1.4 (1.0–1.9)	
	Smoker	20 (10.6)	205 (9.2)	1.4 (0.9–2.4)	
**Education level**	High	41 (22.0	681 (30.4	Ref	<0.001^d^
	Middle	76 (40.9	999 (44.6	1.3 (0.9–1.9	
	Low	69 (37.1	560 (25.0	**2.1 (1.4–3.1**	
**Body mass index (BMI)**	Normal	51 (27.4	725 (32.4	Ref	0.010^d^
	Obese	51 (27.4	446 (19.9	**1.6 (1.1–2.4**	
	Overweight	73 (39.3	1001 (44.7	1.0 (0.7–1.5	
	Underweight	11 (5.9	68 (3.0	**2.3 (1.2–4.6**	
**Kept goats as hobby over past 5 years (46 missing)**	No	173 (96.7	2141 (97.3	Ref	0.624 ^e^
	Yes	6 (3.6	60 (2.7)	1.2 (0.5–2.9)	
**Kept poultry as hobby over past 5 years (93 missing)**	No	149 (85.2	1848 (86.6	1.2 (0.7–1.7)	0.598 ^e^
	Yes	26 (14.9)	290 (13.4)		
**Childhood on farm (24 missing)**	No	112 (61.5	1472 (66.3	Ref	0.193 ^e^
	Yes	70 (38.5)	748 (33.7)	1.2 (0.9–1.7)	
**Grew up outside study area (26 missing)**	No	141 (77.5	1684 (75.9	Ref	0.638 ^e^
	Yes	41 (22.5)	534 (24.1)	0.9 (0.6–1.3)	
**Chronic lung diseases ^b^**	No	138 (74.2	2051 (91.6	Ref	<0.001^d^
	Yes	48 (25.8)	189 (8.4)	**3.8 (2.6–5.4)**	
**Comorbidities ^c^**	No	136 (73.1	1854 (82.8	Ref	0.001 ^d^
	Yes	50 (26.9)	386 (17.2)	**1.8 (1.3–2.5)**	
**Reception of yearly influenza vaccination (19 missing)**	No	98 (53.9	1537 (69.1	Ref	<0.001
	Yes	84 (46.2)	688 (30.9)	**1.9 (1.4–2.6)**	

^a^ Based on Wald chi square test statistic. Variables with a p-value lower than 0.15 were included in the multivariable models in addition to livestock exposure variables

^b^ Variable consists of at least one episode of chronic bronchitis, COPD or asthma recorded in the electronic medical record during the three years preceding the medical examination

^c^ Comorbidity ‘1’ means that participant had at least one (maximum 5) episodes of any hereafter mentioned comorbidity groups recorded in the electronic medical record during the three years preceding the medical examination: chronic cardiovascular disease, cerebrovascular disease, chronic liver disease, chronic nephropathy, malignancies, auto-immune diseases or neurological comorbidities.

^d^ Significant or ^e^ non-significant p-value when adjusting for multiple testing using the Benjamini-Hochberg correction with a false discovery rate of 10%

### General findings regarding associations between livestock farm exposure and pneumonia

The main study focus was on goat and poultry exposure, hence, results presented here are primarily focused on these two species.

Overall, different goat farm exposures were consistently associated with pneumonia in the univariate analysis (Table 3), whereas we found no significant associations for poultry or other animals/ animal farms (Table 3). After adjusting for risk factors, the significant positive associations between goats/ goat farms and pneumonia remained (Table 3). Overall, adjusted odds ratios (aOR) did not vary substantially between the three adjusted models (S3 Table).

**Table 3:** Exposure status and associations from univariate (crude OR) and multiple logistic regression analyses (adjusted OR) between pneumonia and livestock exposure variables. The proportions of cases attributable to respective exposure is indicated by the population attributable fraction (PAF).

		Pneumonia cases (%)	Non-cases (%)	Crude OR	Adjusted OR^a^	PAF^9^
		n = 186	n = 2240	(95% CI)	(95% CI)	(%)
**Number of farms (any type) within 1000m of residence^1^**						
	<6	36 (19.4)	596 (26.6)	Ref	Ref	
	≥6 and <11	88 (47.3)	842 (37.6)	**1.7 (1.2–2.6)**	**1.8 (1.2–2.6)**	20.4
	≥11 (max 32)	62 (33.3)	802 (35.8)	1.3 (0.8–2.0)	1.35 (0.9–2.1)	
**Presence of any type of farm within a certain distance of residence^2^**						
	500m	125 (67.2)	1445 (64.5)	1.1 (0.8–1.6)	1.14 (0.8–1.6)	
	1000m	176 (94.6)	2151 (96.0)	0.7 (0.4–1.4)	0.73 (0.4–1.4)	
**Presence of farm with minimum amount of animals within 500m-intervals of residence^3^**						
**500m^4^**	Goat	11 (5.9)	31 (1.4)	**4.5 (2.2–9.1)**	**4.4 (2.0–9.8)**	4.6
	Poultry	32 (17.2)	322 (14.4)	1.2 (0.8–1.8)	0.95 (0.6–1.5)	
	Pig	60 (32.3)	626 (27.9)	1.2 (1.0–1.7)	1.21 (0.8–1.8)	
	Cattle	100 (53.8)	1176 (52.5)	1.1 (0.8–1.4)	0.91 (0.7–1.3)	
	Horse	50 (26.9)	521 (23.3)	1.2 (0.9–1.7)	1.03 (0.7–1.5)	
	Sheep	12 (6.5)	169 (7.5)	0.9 (0.5–1.6)	0.91 (0.5–1.7)	
**1000m^5^**	Goat	35 (18.8)	229 (10.2)	**2.0 (1.4–3.0)**	**2.0 (1.3–3.1)**	9.5
	Poultry	112 (60.2)	1226 (54.7)	1.3 (0.9–1.7)	1.10 (0.8–1.6)	
	Pig	152 (81.7)	1773 (79.2)	1.2 (0.8–1.7)	1.02 (0.7–1.6)	
	Cattle	174 (93.5)	2110 (94.2)	0.9 (0.5–1.7)	0.64 (0.3–1.3)	
	Horse	143 (76.9)	1599 (71.4)	1.3 (0.9–1.9)	1.28 (0.8–2.0)	
	Sheep	58 (31.2)	701 (31.3)	1.0 (0.7–1.4)	0.93 (0.7–1.3)	
**1500m^6^**	Goat	62 (33.3)	485 (21.7)	**1.8 (1.3–2.5)**	**1.9 (1.4–2.7)**	15.9
	Poultry	156 (83.9)	1890 (84.4)	0.96 (0.6–1.5)	0.74 (0.5–1.2)	
	Pig	183 (98.4)	2172 (97.0)	1.91 (0.6–6.1)	1.65 (0.5–5.6)	
	Cattle	186 (100)	2233 (99.7)	/	/	
	Horse	172 (92.5)	2032 (90.7)	1.26 (0.7–2.2)	1.34 (0.7–2.6)	
	Sheep	102 (54.8)	1357 (60.6)	0.79 (0.6–1.1)	0.78 (0.6–1.1)	
**2000m^7^**	Goat	90 (48.4)	742 (33.1)	**1.9 (1.4–2.6)**	**1.9 (1.4–2.6)**	23.1
	Poultry	175 (94.1)	2040 (91.1)	1.56 (0.8–2.9)	1.35 (0.7–2.7)	
	Pig	186 (100)	2228 (99.5)	/	/	
	Cattle	186 (100)	2240 (100)	/	/	
	Horse	180 (96.8)	2138 (95.4)	1.43 (0.6–3.3)	1.15 (0.5–2.9)	
	Sheep	145 (78)	1776 (79.3)	0.92 (0.6–1.3)	0.88 (0.6–1.3)	
**Distance (quartiles expressed in meters) between residence and closest farm with minimum number of animals**						
**50 goats**	>3490 to 11477	39 (21.0)	568 (25.4)	Ref	Ref	
	>2478 to ≤3490	41 (22.0)	566 (25.3)	1.06 (0.7–1.7)	1.05 (0.7–1.7)	
	>1629 to ≤2478	41 (22.0)	565 (25.2)	1.06 (0.7–1.7)	1.02 (0.7–1.6)	
	99 to ≤ 1629	65 (34.9)	541 (24.2)	**1.8 (1.2–2.7)**	**1.8 (1.2–2.7)**	15.3
**250 poultry**	>1296 to 4145	46 (24.7)	561 (25.0)	Ref	Ref	
	>923to ≤1296	33 (17.7)	573 (25.6)	0.70 (0.4–1.1)	0.69 (0.4–1.1)	
	>644 to ≤923	47(25.3)	557 (24.9)	1.03 (0.7–1.6)	1.03 (0.7–1.6)	
	39 to ≤644	60(32.2)	549 (24.5)	1.33 (0.9–2.0)	1.38 (0.9–2.1)	
**Number of animals within 1000m of the residence^8^**						
**Goats (no tertiles)**	0	133 (71.5)	1857 (82.9)	Ref	Ref	
	>0 to ≤50	18 (9.7)	155 (6.9)	1.62 (0.97–2.72)	1.54 (0.88–2.69)	3.4
	>50	35 (18.8)	228 (10.2)	**2.14 (1.44–3.19)**	**1.67 (1.06–2.63)**	7.5
**Poultry (tertiles)**	0	55 (29.6)	794 (35.4)	Ref	Ref	
	>0 to ≤28250	64 (34.4)	689 (30.8)	1.34 (0.92–1.95)	1.12 (0.74–1.69)	
	>28250	67 (36.0)	757 (33.8)	1.28 (0.88–1.85)	1.09 (0.70–1.69)	

^a^ Adjusted for age and gender

^1^ Any type of animal farm (main farming category, expressed in tertiles)

^2^ Main farm category (any type of animal farm) as registered in the livestock database

^3^ Minimum amount of animals: 50 goats, 250 poultry, 25 pigs, 5 cattle, 5 horses, 50 sheep

^4^ Also adjusted for presence of other farms within 500m with a minimum number of animals

^5^ Also adjusted for presence of other farms within 1000m with a minimum number of animals

^6^ Also adjusted for presence of other farms within 1500m with a minimum number of animals

^7^ Also adjusted for presence of other farms within 2000m with a minimum number of animals

^8^ Also adjusted for number of other animals within 1000m

^9^ Population attributable fraction based on model A

In multivariable logistic regression analyses, none of the other determinants adjusted for remained an independent risk factor with exception of chronic lung diseases.

#### Association between seropositivity against Coxiella burnetii and pneumonia

For 2,358 of the 2,426 participants (97%), serological results for *Coxiella burnetii* were available. Of these, 146 were seropositive (6.2%). Among those seropositive, 13 (8.9%) also have had pneumonia in the previous three years. However, no statistically significant association between having had pneumonia and being seropositive for *Coxiella burnetii* (dichotomous variable) was found (Table 4). Results did not change when using the three-category serostatus for *C*. *burnetii* (negative, borderline or positive; p-value>0.7).

**Table 4:** Characteristics of the study population for Q fever related exposures.

Characteristic		Pneumonia cases	Non-cases	Crude odds ratio	p-value^a^
		n (%)	n (%)	(95% CI)	
**Serostatus for Coxiella burnetii (Q-fever) (68 missing**	Negative	165 (92.7)	2047 (93.9)	Ref	>0.5 ^d^
	Positive & borderline combined	13 (7.3)	133 (6.1)	1.2 (0.7–2.2)	
**Serostatus for *Coxiella burnetii*^b^**	Negative	165 (94.8)	2047 (96.1)	Ref	>0.4 ^d^
	Only positive	9 (5.2)	84 (3.9)	1.3 (0.7–2.7)	
**Living within 1500m of Q-fever positive farm^c^**	No	177 (95.2)	2120 (94.6)	Ref	>0.7 ^d^
	Yes	9 (4.8)	120 (5.4)	0.9 (0. 5–1.8)	
**Living within 2000m of Q-fever positive farm**	No	159 (85.5)	1996 (89.1)	Ref	>0.1 ^d^
	Yes	27 (14.5)	244 (10.9)	1.4 (0.9–2.1)	

^a^ Based on Wald chi square test statistic. Variables with a p-value lower than 0.15 were included in the multivariable models in addition to livestock exposure variables

^b^ For the sensitivity analysis we excluded participants with borderline serological results.

^c^ No pneumonia cases lived within 500 or 1000m of a Q-fever positive goat farm (defined as testing positive for *Coxiella burnetii* in bulk milk or having had abortion storms during the Q fever epidemic)

^d^ non-significant when adjusting for multiple testing using the Benjamini-Hochberg correction with a false discovery rate of 10%

#### Exposure to Q fever-positive farms

When investigating associations between goat farms that tested positive for Q fever during the epidemic and pneumonia, we found that no Q fever-positive farm was located within 500 or 1000m of the residence of a case. The number of pneumonia cases that lived within 1500 or 2000m to Q fever-positive farms was low (Table 4). Neither living within 1500m, nor 2000m of a Q fever positive farm was associated with pneumonia (Table 4).

Distance to farms with Q fever-positive bulk milk samples in 2010 was similar for cases and non-cases (median distance 3777m, IQR: 2924–5611 versus 4023m, IQR 2860–6108; p-value 0.133). Median distance between residence and farms with Q fever-induced abortions was significantly shorter for cases (median distance 7042m, IQR: 3233–12,550 versus 9760, IQR 3770–12,748; p-value 0.0013). However, distances to Q fever-positive farms were still substantially larger compared to distances to farms with ≥50 goats, as described above.

#### Presence of farms within 500m increments to residence

The presence of at least one goat farm (with a minimum of 50 goats) within 500m increments of the home address (ranging from 500m to 2000m), was significantly positively associated with pneumonia, whereas such associations were not, or not consistently, found for other types of livestock farms (Table 3). Adjusted ORs for presence of goat farm increased in magnitude the closer to the residence goat farms were located (Table 3); a trend analysis confirmed the observed dose-response relationship (p-value <0.001). This was also observed when an adjustment was made for the presence of other animal farms than goat farms.

#### Distance between farms and residence

Pneumonia cases lived significantly closer to farms with ≥50 goats, compared to non-cases (median distance 2090m, IQR 1222–3291 versus 2501m, IQR 1663-3511m; p-value 0.0015). A shorter distance to goat farms with at least 50 goats (Q4, ~100 to 1,600m) compared to a longer one (Q1, ~3,500 to 11,500m) was significantly associated with pneumonia (Table 3).

Poultry farms with a minimum amount of 250 birds were located closer to cases than non-cases (median distance 794m, IQR 560–1291 versus 936m, 647–1298; p-value 0.041). A shorter distance to poultry farms with at least 250 birds, was also positively associated with pneumonia compared to a larger distance (Q4 vs. Q1), but associations were not statistically significant (Table 3).

#### Number of animals within 1000m of the residence

The presence of more than 50 goats within 1000m of the residence was significantly associated with pneumonia (aOR 1.7, 95% CI 1.1–2.6), compared to having no goats close to the home (Table 3). Although number of poultry within 1000m of the residence was substantially higher, no association with pneumonia was found (Table 3).

#### Population attributable fraction

The number of pneumonia cases exposed to goat farms ranged from 11 (living within 500m) to 90 (living within 2000m of a goat farm; Table 3). The population attributable fraction (PAF; based on model A), i.e. the fraction of cases that could be avoided if the exposure to goat farms were to be removed, was low (4.6%) for those living within 500m of a goat farm. For a 1000m to 2000m-perimeter, the PAF gradually increased from 9.6% to 23.1%, respectively (Table 3).

### Sensitivity analyses using EMR recorded pneumonia as outcome variable

When repeating the analyses using pneumonia based on the EMR only as outcome, i.e. using ‘Having had a pneumonia episode registered in the EMR in the three years preceding the medical examination’, the associations between goat farm exposure and pneumonia remained robust and the magnitude was comparable to when the combined pneumonia outcome was used (S4 Table). When using the EMR-based pneumonia variable, associations between pneumonia and presence of a poultry farm (with at least 250 birds) within 1000m of the residence reached statistical significance (OR adjusted for age and sex: 1.7, 95% CI 1.1–2.7). For other 500m-intervals (500m, 1500m, 2000m), no statistically significant association with poultry farm presence was found (S4 Table).

### Non-response analyses

Participants who were older, female and lived closer to a livestock farm were more likely to fill in the short questionnaire, as well as more likely to participate in the medical examination (data not shown). Also, people with a goat farm present within 1000m of their home (irrespective of minimum number of goats) were more likely to participate in the medical examination. However, in all subpopulations (total population, individuals who responded to the short questionnaire, all individuals invited to the medical examination, individuals who took part in the medical examination) [11], we found consistent associations between pneumonia (as registered in the EMR) and presence of a goat farm within 1000m of the home. ORs adjusted for age and gender ranged from 2.1 (95% CI 1.6–2.8) for the total population to 2.4 (95% CI 1.3–4.5) for those who participated in the medical examination.

## Discussion

In this study in non-farming adults from the general population, we found that the presence of goat farms near the home was strongly positively associated with pneumonia, with increasing odds the closer the residence was located to the farm. Remarkably, the present study investigated the occurrence of pneumonia between 2012 and 2015, whereas similar results were found in a study conducted during a Q fever outbreak in 2009 [8]. A positive test for antibodies against *Coxiella burnetii* was not associated with pneumonia. The magnitude of the association between the presence of a poultry farm within 1000m and pneumonia was comparable to previous findings when using EMR-recorded pneumonia as an outcome. No associations between other animal farms/ types and pneumonia was found.

The finding that goat farms still pose a risk factor for pneumonia in a period where the Q-fever incidence among humans had dropped to pre-epidemic levels–i.e. 63 notifications with onset of illness in 2012, 20 in 2013, and 26 in 2014 –was unexpected and we explored several explanations for the association between pneumonia and proximity to goat farms. One explanation is that people who lived closer to goat farms were more likely to participate in this study compared to those living further away, which could possibly introduce selection bias. However, as the non-response analyses resulted in comparable associations between presence of goat farms and pneumonia in all three subpopulations, self-selection bias seems unlikely.

Bias in self-reported pneumonia is unlikely to be the explanation for the association as they were also found with EMR-registered pneumonia. For data obtained through the questionnaire, we attempted to avoid misclassification bias–meaning that, for instance similar clinical presentations, such as acute bronchitis/ bronchiolitis might falsely be classified as pneumonia–by only including cases who reported pneumonia and who reported having had their diagnosis confirmed by a physician. When comparing reports of self-reported, physician diagnosed pneumonia with pneumonia records from the EMR, 121 (65%) participants had a record of pneumonia in both sources. For 65 (35%) participants pneumonia was only recorded in the questionnaire but not in the EMR. The data from the EMR represented data from a GP primary care network, hence, we could not check whether the 35% that stated having had pneumonia in the questionnaire were diagnosed by a specialist or in a hospital, which would explain why no pneumonia was recorded in the EMR. In the Netherlands, about 80% of pneumonia cases are managed in primary care [16]. This would suggest that 15% of the pneumonia cases in our study could be misclassified, which could overestimate odds ratios by introducing differential misclassification bias, if cases who lived closer to goat farms falsely reported having had pneumonia. Among the 65 cases who did not have an EMR entry for pneumonia in the three years preceding the medical examination, 13 had an entry for acute bronchitis/bronchiolitis (20%), a condition that can present with similar symptoms as pneumonia [17].

A previous study conducted between April 2008 and March 2009 showed that cases with Q fever pneumonia were more likely to live in a region with a high goat density or close to sheep [18]. However, in the present study, we found no positive association between pneumonia and being seropositive for *Coxiella burnetii*, or living within 500 or 1000m of a Q fever-positive farm, respectively, which makes it unlikely that the associations presented here are primarily attributable to this zoonosis. A limitation of our study was that no information was available concerning causative pathogens or season in which pneumonia occurred, neither did we conduct serological analyses for other pathogens. A previous study in a large hospital in the province of North-Brabant, investigated which pathogens are most frequently detectable in patients with community-acquired pneumonia (CAP) [19]. This prospective observational study conducted between November 2007 and January 2010, coinciding with the Q fever epidemic, showed, that *Streptococcus pneumoniae* was most frequently identified bacterium (22%), followed by *Coxiella burnetii* (14%), *Mycoplasma pneumonia* (6%) and *Haemophilus influenzae* (6%). Although these results give interesting insights in the etiology of CAP, the authors stressed that continuous microbiological surveillance in combination with clinical symptoms is needed to be able to monitor seasonal variations and allow extrapolation to other years [19]. More recently, another group studied CAP in four Dutch hospital cohorts covering the periods 1998–2000 and 2004–2010, and found that atypical microorganisms, such as *Legionella* species, *Coxiella burnetii*, *Mycoplasma pneumoniae* and *Chlamydia* species were predominantly detected during the non-respiratory season defined as week 20 to week 39 (40.4%), as compared to the respiratory season (12.4%; week 40 to week 19) [20]. Awareness of the importance of seasonal patterns and including exposure to animals in medical history can help to guide clinicians in targeted testing for atypical pathogens outside the respiratory season. Whether atypical/ other zoonotic pathogens might also play a role in people living in a goat-dense area presenting with CAP could be addressed in future studies.

Besides infectious causes, non-infectious causes, such as mold, inhalable dust and endotoxins, might also play a role in explaining the associations between animal farms and pneumonia. Various experiments demonstrated that inhalation of fine dusts and endotoxins can increase susceptibility for infection with common human pathogens [21, 22] and that inhalation of (urban) particulate matter can lead to pneumonia in humans, through increased adhesion of *S*. *pneumoniae* to human airway epithelium [23]. Exposure to dust was also identified as a risk factor for CAP among professionals exposed to different working conditions [24], however measurements of such non-infectious exposures were not yet available in this study which constitutes a limitation. To analyze the relation between CAP and the composition of dust emission from goat farms, air filter techniques, as described previously, could be utilized in future research [25]. Dairy goats in the Netherlands are kept in deep litter stables with partly open roofs or walls. The deep litter husbandry system is characterized by topping up soiled litter, such as straw or hay, with fresh litter such as straw or hay every few days. When the layer of litter becomes too high, manure is removed and stored on a dunghill before being spread on farmland as a fertilizer [26]. A small study investigating the contribution of different dust sources to dust mass transmitted via air showed, that straw used for bedding contributed more than 50% to fine and coarse dust emissions. However, compared to cow farms, goat farms emit twelve times less total dust per kg metabolic weight [25], suggesting that dust emitted from goat farms might not be the most evident explanation of our results.

Although dust and endotoxins might not contribute substantially to explaining the observed association between goat farms and pneumonia, it might be relevant in explaining part of the association between pneumonia and poultry farms (S3 and S4 Tables). A comprehensive study from Denmark studied emissions of inhalable dust and endotoxins between different farming types and seasons and found, that compared to other farming types, poultry and pig farmers were exposed to the highest levels of fine dust and endotoxins [27]. That poultry farm emissions might also be relevant for the health of neighboring residents was shown in a Dutch study, which found elevated endotoxin levels 250m downwind from poultry farms [7].

Another interesting future research avenue is to investigate the indirect effects of non-infectious agents on the risk of CAP by studying the composition of the human upper respiratory tract microbiome in residents living close to goat farms. A recent study hypothesized that exposure to farm emissions may result in changes of the composition of the upper airway microbiome, which might lead to commensals, such as *S*. *pneumoniae*, to become pathogens. An association was found between living close to poultry farms and CAP that possibly resulted from alterations of the oropharyngeal microbiota composition. As this was the first study that showed such an association, the researchers stressed that these findings need to be replicated in larger studies [28].

The relatively small sample size constitutes a limitation of our study. In the study by Smit et al. [8], the association between presence of a poultry farm within 1km of the home address and pneumonia was relatively low (OR 1.25, 95% CI 1.06–1.47). It is conceivable that the current study was underpowered to detect an association with poultry exposure when using the combined pneumonia outcome, although results were close to statistical significance. When using EMR-based pneumonia as outcome, a significant association with poultry exposure within 1000m was found. Similarly, to above, zoonotic pathogens might play a role in those presenting with CAP and living close to poultry. For instance, *Chlamydia psittaci*, a zoonotic bacterium associated with poultry causes psittacosis in humans. In CAP-etiological studies, this bacterium is often not considered or only incidentally isolated. However, a study from the Netherlands identified 7/147 (4.8%) CAP patients with psittacosis [18, 19, 29], suggesting that the role of *C*. *psittaci* is more important than often assumed.

The research center Heeswijk-Dinther had the highest prevalence of pneumonia (16.3%, Table 1), which also coincided with the highest seroprevalence against *Coxiella burnetii* (10%; average seroprevalence: 6%) [10]. We examined goat farm density per research center and found that exposure to farms with ≥50 goats (defined as presence within 500m increments of the residence) for individuals examined at Heeswijk-Dinther was highest compared to other study centers. However, in the current study, no association between Q fever and pneumonia was found (Table 4). As older age is as a risk factor for pneumonia, we examined whether a higher percentage of older people lived in Heeswijk-Dinther compared to other study centers. However, although we found slight, nevertheless significant, differences between the centers (Kruskall Wallis test, Chi^2^ value 21.1, p-value = 0.03), the median score for age was generally lower for Heeswijk-Dinther, compared to other study centers (data not shown), thereby dismissing this hypothesis. As there is currently no good explanation why the proportion of pneumonia cases was higher in Heeswijk-Dinther compared to other study centers, this question could be addressed in future research.

In conclusion, in this study we showed that living in the vicinity of goat farms still constitutes a risk factor for pneumonia. As it was surprising that the association between goat exposure and pneumonia was still found in a period where Q fever in humans has only occurred sporadically, however, Q fever itself seemed an unlikely explanation of the findings, future research should be directed to studying the role of alternative infectious and non-infectious causes to be able to assess possible implications for public health and provide evidence-based recommendations. To shed light on the contribution of atypical pathogens other than *Coxiella burnetii [20]* to the CAP burden near goat and poultry farms, molecular diagnostics for *Legionella* species, *Mycoplasma pneumoniae* and *Chlamydia* species should be considered in CAP patients from these regions. In addition, the role of the composition of the human upper airway microbiome [28] in people with CAP living close to goat farms should be investigated in future research.

## Supporting information

S1 Fig Distribution of number of goats (x-axis) within 1000m of the residence. Due to the highly skewed distribution, the construction of tertiles was not possible (minimum: 0, Q1: 0, median: 0, Q3: 0, maximum: 5015). To analyse associations between pneumonia and number of goats within 1000m, we therefore created a variable with three categories (0: 0 goats, 1: >0 and ≤50 goats, 2: >50 goats). The cut-off of 50 was chosen based on a threshold applied during the compulsory vaccination campaign during the Q fever epidemic.
(TIF)

S1 Table English translation of questions from the complete Dutch questionnaire used for analyses described in this article. A full version of the Dutch questionnaire is appended.
(DOCX)

S2 Table List of comorbidities regarded as a risk factor for pneumonia. A person was counted as ‘Having had any comorbidity’, if at least one of the listed comorbidities was recorded in the electronic medical record in the three years preceding the medical exam.
(DOCX)

S3 Table Associations from univariate (crude OR) and multiple logistic regression analyses between pneumonia and livestock exposure variables. For the multiple logistic regression analysis, we ran three different models in which we added potential confounders consecutively (Models A-C).
(DOCX)

S4 Table Results from sensitivity analyses using ‘Having had a pneumonia episode registered in the EMR (ICPC code R81) in the three years preceding the medical exam’ (i.e. between 2012 and 2014/15) as the outcome. (DOCX)

S1 File Full version of the original Dutch questionnaire. (PDF)
