# Supplementary figures and images for: Livestock-associated risk factors for pneumonia in an area of intensive animal farming in the Netherlands
Source: PLoS One. 2017 Mar 31;12(3):e0174796. doi: 10.1371/journal.pone.0174796 (PMC5376295; doi:10.1371/journal.pone.0174796)

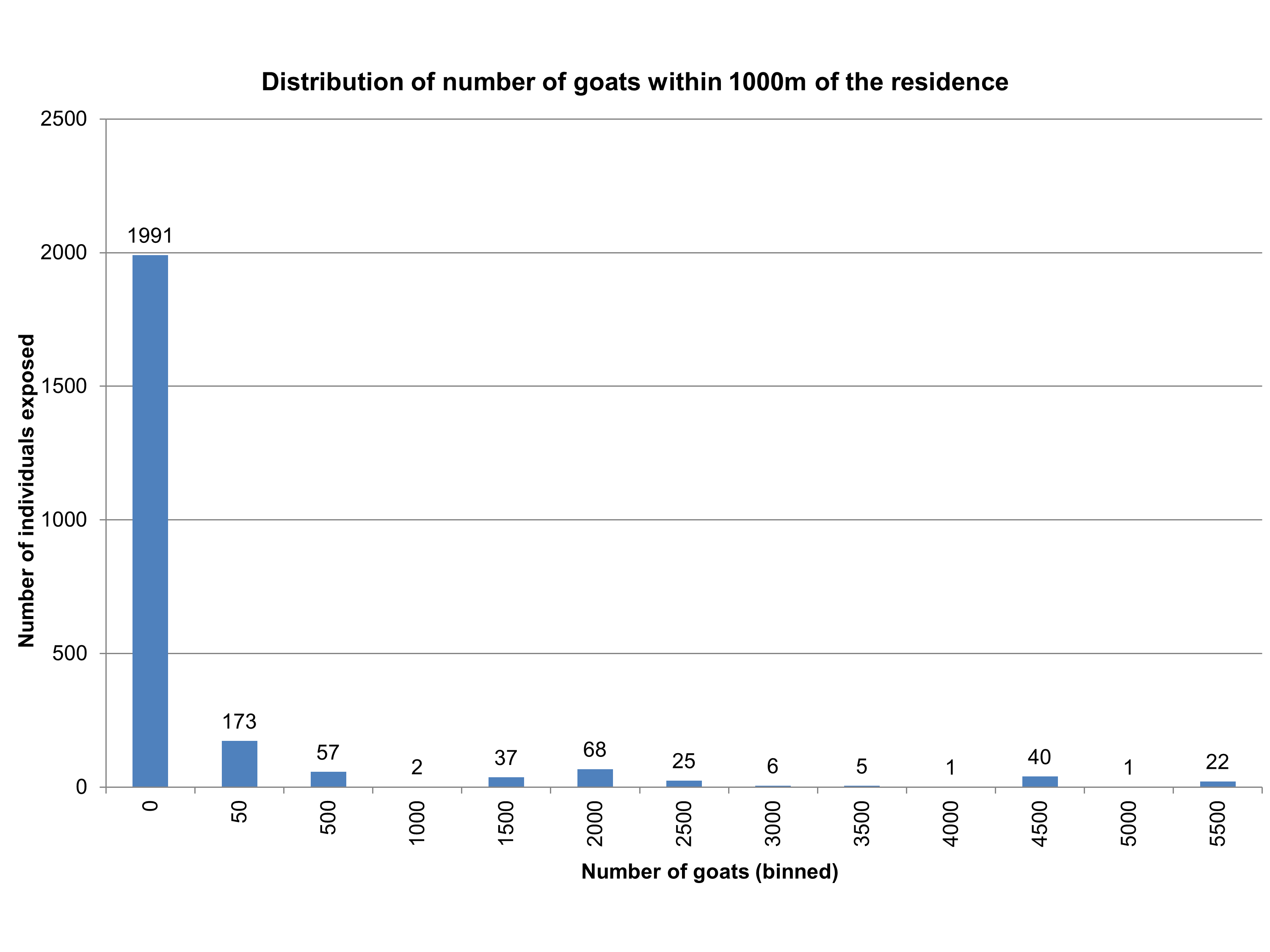

Supplement: S1 Fig — Due to the highly skewed distribution, the construction of tertiles was not possible (minimum: 0, Q1: 0, median: 0, Q3: 0, maximum: 5015). To analyse associations between pneumonia and number of goats within 1000m, we therefore created a variable with three categories (0: 0 goats, 1: >0 and ≤50 goats, 2: >50 goats). The cut-off of 50 was chosen based on a threshold applied during the compulsory vaccination campaign during the Q fever epidemic. (TIF) [file pone.0174796.s001.tif]
