# Supplementary material for: Livestock-associated risk factors for pneumonia in an area of intensive animal farming in the Netherlands
Source: PLoS One. 2017 Mar 31;12(3):e0174796. doi: 10.1371/journal.pone.0174796 (PMC5376295; doi:10.1371/journal.pone.0174796)
Supplement: S1 Table — A full version of the Dutch questionnaire is appended. (DOCX) [file pone.0174796.s002.docx]

| **Dutch** | | **English** | |
| --- | --- | --- | --- |
| **Question** | **Answer option(s)** | **Question** | **Answer option(s)** |
| Wat is uw geboortedatum? | dd-mm-jjjj | What is your date of birth? | dd-mm-yyyy |
| Wat is uw geslacht? | Man | What is your gender? | male |
|  | Vrouw |  | female |
| Heeft u (ooit) sigaretten, sigaren en/of pijpen gerookt? | Nee | Have you (ever) smoked cigarettes and/ or pipes? | No |
|  | Ja, ik heb vroeger gerookt, maar ik ben └────┘ jaar geleden gestopt |  | Yes, I smoked in the past, but stopped └────┘years ago |
|  | Ja, ik rook (nog steeds) |  | Yes, I (still) smoke |
| Heeft u **de afgelopen 5 jaar** landbouwhuisdieren gehouden als hobby? | Ja | Did you keep farm animals for a hobby during the **past five years**? | Yes |
|  | Nee |  | No |
| Welke soort landbouwhuisdier(en) heeft u **de afgelopen 5 jaar** als  hobby gehouden? | Geit | Which kind of farm animal(s) did you keep for a hobby during the **past 5 years**? | Goat |
|  | Kip, kalkoen, eend  of gans |  | Chicken, turkey, duck or goose |
| Heeft u als kind (tot 18 jaar) op een boerderij met dieren gewoond? | Nee | Did you live on an animal farm (until the age of 18)? | No |
|  | Ja, namelijk van leeftijd └────┘ (*jaar oud*)  tot leeftijd └────┘ (*jaar oud*) |  | Yes, from age └────┘to age└────┘ *(in years)* |
| In welke gemeente/woonplaats heeft u het grootste deel van uw jeugd (tot 18 jaar)  doorgebracht? | open vraag | In which municipality/ place did you spend most of your youth (until the age of 18)? | open question |
| Neemt u deel aan de jaarlijkse griepvaccinatie (griepprik)? | Ja | Do you participate in the yearly influenza vaccination campaign? | Yes |
|  | Nee |  | No |
| Uit hoeveel personen bestaat uw huishouden, naast u zelf?  (*meerdere antwoorden mogelijk*) | Ik woon alleen | How many persons does your household consist of, besides yourself? *(more answers possible)* | I live alone |
|  | Met partner |  | I live with my partner |
|  | Inwonende ouders of andere nog niet genoemde volwassenen: └────┘(*aantal*) |  | I live with my parents or other adults that have not yet been mentioned: └────┘(*number*) |
|  | Kinderen van 18 jaar of ouder: └────┘(*aantal*) |  | Children of 18 years or older: └────┘(*number*) |
|  | Kinderen 4 t/m 17 jaar: └────┘(*aantal*) |  | Children between 4 and 17 years old: : └────┘(*number*) |
|  | Kinderen jonger dan 4 jaar: └────┘(*aantal*) |  | Children younger than 4 years: : └────┘(*number*) |
