# Supplementary material for: Livestock-associated risk factors for pneumonia in an area of intensive animal farming in the Netherlands
Source: PLoS One. 2017 Mar 31;12(3):e0174796. doi: 10.1371/journal.pone.0174796 (PMC5376295; doi:10.1371/journal.pone.0174796)
Supplement: S2 Table — A person was counted as ‘Having had any comorbidity’, if at least one of the listed comorbidities was recorded in the electronic medical record in the three years preceding the medical exam. (DOCX) [file pone.0174796.s003.docx]

| **Comorbidity group** | **Condition** | **ICPC code** | **References** |
| --- | --- | --- | --- |
| Cerebrovascular disease | Stroke | K90 | [1-3] |
|  | Transient ischemic heart disease | K89 |  |
|  | Dementia | P70 |  |
| Chronic cardiovascular disease | Other/ chronic ischemic heart diseases | K76 | [2-4] |
|  | Decompensatio cordis (Heart failure) | K77 |  |
|  | Atrial fibrillation | K78 |  |
|  | Paroxysmal tachycardia | K79 |  |
|  | Extrasystoles | K80 |  |
|  | Cor pulmonale | K82 |  |
|  | Non-rheumatic heart valve conditions | K83 |  |
|  | Other heart diseases | K84 |  |
| Chronic liver disease | Viral hepatitis | D72 | [2, 3] |
|  | Cirrhosis | D97 |  |
| Chronic nephropathy | Glomerulonephritis | U88 | [2, 3] |
|  | Other diseases of the urinary tract | U99 |  |
| Autoimmune diseases | Crohn’s disease (colitis and ulceritis) | D94 | [2, 3, 5, 6] |
|  | Rheumatoid arthritis/ related diseases | L88 |  |
| Malignancy | Lung cancer | R84 | [1, 2, 4] |
|  | Breast cancer | X76 |  |
|  | Prostate cancer | Y77 |  |
|  | Gastric cancer | D74 |  |
| Neurological comorbidity | Parkinson’s disease | N87 | [2, 4, 7] |
|  | Epilepsy | N88 |  |
| Chronic lung diseases | Chronic bronchitis/ bronchiectasis | R91 | [2, 4] |
|  | Chronic obstructive pulmonary disease (COPD) | R95 |  |
|  | Asthma | R96 |  |

**References**

1. Vila-Corcoles A, Aguirre-Chavarria C, Ochoa-Gondar O, de Diego C, Rodriguez-Blanco T, Gomez F, et al., Influence of chronic illnesses and underlying risk conditions on the incidence of pneumococcal pneumonia in older adults*.* Infection, 2015. **43**(6): p. 699-706.

2. Torres A, Peetermans WE, Viegi G, and Blasi F, Risk factors for community-acquired pneumonia in adults in Europe: a literature review*.* Thorax, 2013. **68**(11): p. 1057-65.

3. Vinogradova Y, Hippisley-Cox J, and Coupland C, Identification of new risk factors for pneumonia: population-based case-control study*.* Br J Gen Pract, 2009. **59**(567): p. e329-38.

4. Almirall J, Bolibar I, Serra-Prat M, Roig J, Hospital I, Carandell E, et al., New evidence of risk factors for community-acquired pneumonia: a population-based study*.* Eur Respir J, 2008. **31**(6): p. 1274-84.

5. Shea KM, Edelsberg J, Weycker D, Farkouh RA, Strutton DR, and Pelton SI, Rates of pneumococcal disease in adults with chronic medical conditions*.* Open Forum Infect Dis, 2014. **1**(1): p. ofu024.

6. Wotton CJ and Goldacre MJ, Risk of invasive pneumococcal disease in people admitted to hospital with selected immune-mediated diseases: record linkage cohort analyses*.* J Epidemiol Community Health, 2012. **66**(12): p. 1177-81.
