# Supplementary material for: Livestock-associated risk factors for pneumonia in an area of intensive animal farming in the Netherlands
Source: PLoS One. 2017 Mar 31;12(3):e0174796. doi: 10.1371/journal.pone.0174796 (PMC5376295; doi:10.1371/journal.pone.0174796)
Supplement: S3 Table — For the multiple logistic regression analysis, we ran three different models in which we added potential confounders consecutively (Models A-C). (DOCX) [file pone.0174796.s004.docx]

|  |  | Pneumonia cases (%)  n=186 | Non-cases  (%)  n=2240 | Crude OR  (95%CI) | Adjusted OR  Model A^a^  (95%CI) | Adjusted OR  Model B^b^  (95%CI) | Adjusted OR  Model C^c^  (95%CI) | PAF^9^  (%) |
| --- | --- | --- | --- | --- | --- | --- | --- | --- |
| Number of farms (any type) within 1000m of residence^1^ | | | | | | | | |
|  | <6 | 36 (19.4) | 596 (26.6) | Ref | Ref | Ref | Ref |  |
|  | ≥6 and <11 | 88 (47.3) | 842 (37.6) | **1.73 (1.16-2.59)** | **1.76 (1.17-2.63)** | **1.78 (1.19-2.67)** | **1.82 (1.20-2.74)** | 20.4 |
|  | ≥11 (max 32) | 62 (33.3) | 802 (35.8) | 1.28 (0.84-1.96) | 1.35 (0.88-2.06) | 1.37 (0.89-2.10) | 1.40 (0.91-2.17) |  |
| Presence of any type of farm within a certain distance of the residence^2^ | | | | | | | | |
|  | 500m | 125 (67.2) | 1445 (64.5) | 1.13 (0.82-1.55) | 1.14 (0.83-1.57) | 1.17 (0.85-1.62) | 1.22 (0.88-1.69) |  |
|  | 1000m | 176 (94.6) | 2151 (96.0) | 0.73 (0.37—1.42) | 0.73 (0.37-1.43) | 0.76 (0.39-1.51) | 0.80 (0.40-1.61) |  |
| Presence of animal farm with minimum amount of animals within 500m-increments of the residence^3^ | | | | | | | | |
| 500m^4^ | Goat | 11 (5.9) | 31 (1.4) | **4.48 (2.21-9.06)** | **4.38 (1.96-9.80)** | **4.34 (1.92-9.84)** | **3.69 (1.61-8.47)** | 4.6 |
|  | Poultry | 32 (17.2) | 322 (14.4) | 1.24 (0.83-1.84) | 0.95 (0.59-1.51) | 0.93 (0.58-1.49) | 0.91 (0.56-1.46) |  |
|  | Pig | 60 (32.3) | 626 (27.9) | 1.23 (0.89-1.69) | 1.21 (0.83-1.78) | 1.20 (0.82-1.77) | 1.26 (0.85-1.85) |  |
|  | Cattle | 100 (53.8) | 1176 (52.5) | 1.05 (0.78-1.42) | 0.91 (0.65-1.28) | 0.95 (0.67-1.34) | 0.96 (0.68-1.36) |  |
|  | Horse | 50 (26.9) | 521 (23.3) | 1.21 (0.87-1.70) | 1.03 (0.69-1.53) | 1.02 (0.69-1.53) | 1.04 (0.70-1.56) |  |
|  | Sheep | 12 (6.5) | 169 (7.5) | 0.85 (0.46-1.55) | 0.91 (0.49-1.68) | 0.90 (0.48-1.68) | 0.93 (0.50-1.76) |  |
| 1000m^5^ | Goat | 35 (18.8) | 229 (10.2) | **2.04 (1.38-3.01)** | **2.01 (1.32-3.05)** | **2.02 (1.32-3.08)** | **1.97 (1.28-3.03)** | 9.5 |
|  | Poultry | 112 (60.2) | 1226 (54.7) | 1.25 (0.92-1.70) | 1.10 (0.78-1.57) | 1.10 (0.77-1.57) | 1.08 (0.75-1.55) |  |
|  | Pig | 152 (81.7) | 1773 (79.2) | 1.18 (0.80-1.73) | 1.02 (0.65-1.60) | 1.02 (0.65-1.60) | 1.09 (0.69-1.73) |  |
|  | Cattle | 174 (93.5) | 2110 (94.2) | 0.89 (0.49-1.65) | 0.64 (0.32-1.31) | 0.67 (0.33-1.36) | 0.66 (0.32-1.37) |  |
|  | Horse | 143 (76.9) | 1599 (71.4) | 1.33 (0.94-1.90) | 1.28 (0.84-1.97) | 1.29 (0.84-1.98) | 1.31 (0.84-2.03) |  |
|  | Sheep | 58 (31.2) | 701 (31.3) | 1.00 (0.72-1.37) | 0.93 (0.67-1.31) | 0.92 (0.66-1.30) | 0.93 (0.66-1.31) |  |
| 1500m^6^ | Goat | 62 (33.3) | 485 (21.7) | **1.81 (1.31-2.49)** | **1.91 (1.38-2.67)** | **1.87 (1.34-2.62)** | **1.91 (1.36-2.68)** | 15.9 |
|  | Poultry | 156 (83.9) | 1890 (84.4) | 0.96 (0.64-1.45) | 0.74 (0.46-1.18) | 0.75 (0.46-1.22) | 0.72 (0.44-1.17) |  |
|  | Pig | 183 (98.4) | 2172 (97.0) | 1.91 (0.60-6.13) | 1.65 (0.49-5.62) | 1.64 (0.48-5.61) | 1.81 (0.52-6.28) |  |
|  | Cattle | 186 (100) | 2233 (99.7) | / | / | / | / |  |
|  | Horse | 172 (92.5) | 2032 (90.7) | 1.26 (0.72-2.21) | 1.34 (0.70-2.59) | 1.36 (0.70-2.64) | 1.51 (0.77-2.96) |  |
|  | Sheep | 102 (54.8) | 1357 (60.6) | 0.79 (0.59-1.07) | 0.78 (0.57-1.06) | 0.79 (0.58-1.07) | 0.77 (0.56-1.05) |  |
| 2000m^7^ | Goat | 90 (48.4) | 742 (33.1) | **1.89 (1.40-2.56)** | **1.91 (1.41-2.59)** | **1.86 (1.37-2.53)** | **1.89 (1.39-2.59)** | 23.1 |
|  | Poultry | 175 (94.1) | 2040 (91.1) | 1.56 (0.83-2.92) | 1.35 (0.68-2.66) | 1.37 (0.69-2.72) | 1.38 (0.69-2.76) |  |
|  | Pig | 186 (100) | 2228 (99.5) | / | / | / | / |  |
|  | Cattle | 186 (100) | 2240 (100) | / | / | / | / |  |
|  | Horse | 180 (96.8) | 2138 (95.4) | 1.43 (0.62-3.30) | 1.15 (0.47-2.85) | 1.14 (0.46-2.85) | 1.21 (0.48-3.06) |  |
|  | Sheep | 145 (78) | 1776 (79.3) | 0.92 (0.64-1.33) | 0.88 (0.61-1.27) | 0.89 (0.62-1.29) | 0.87 (0.60-1.27) |  |
| Distance (quartiles expressed in meters) between residence and closest farm with minimum number of animals (quartiles, expressed in meters) | | | | | | | | |
| 50 goats | >3490 to 11477 | 39 (21.0) | 568 (25.4) | Ref | Ref | Ref | Ref |  |
|  | >2478 to ≤3490 | 41 (22.0) | 566 (25.3) | 1.06 (0.67-1.66) | 1.05 (0.67-1.66) | 1.07 (0.68-1.69) | 1.11 (0.70-1.77) |  |
|  | >1629 to ≤2478 | 41 (22.0) | 565 (25.2) | 1.06 (0.67-1.66) | 1.02 (0.65-1.62) | 1.05 (0.66-1.66) | 1.07 (0.67-1.70) |  |
|  | 99 to ≤ 1629 | 65 (34.9) | 541 (24.2) | **1.75 (1.16-2.65)** | **1.78 (1.17-2.70)** | **1.77 (1.17-2.69)** | **1.85 (1.21-2.83)** | 15.3 |
| 250 poultry | >1296 to 4145 | 46 (24.7) | 561 (25.0) | Ref | Ref | Ref | Ref |  |
|  | >923to ≤1296 | 33 (17.7) | 573 (25.6) | 0.70 (0.44-1.12) | 0.69 (0.43-1.09) | 0.70 (0.44-1.12) | 0.71 (0.45-1.14) |  |
|  | >644 to ≤923 | 47(25.3) | 557 (24.9) | 1.03 (0.67-1.57) | 1.03 (0.67-1.57) | 1.06 (0.69-1.62) | 1.06 (0.69-1.64) |  |
|  | 39 to ≤644 | 60(32.2) | 549 (24.5) | 1.33 (0.89-1.99) | 1.38 (0.92-2.07) | 1.39 (0.92-2.08) | 1.43 (0.94-2.15) |  |
| Number of animals within 1000m of the residence^8^ | | | | | | | | |
| Goats  (no tertiles) | 0 | 133 (71.5) | 1857 (82.9) | Ref | Ref | Ref | Ref |  |
|  | >0 to ≤50 | 18 (9.7) | 155 (6.9) | 1.62 (0.97-2.72) | 1.54 (0.88-2.69) | 1.58 (0.90-2.77) | 1.44 (0.81-2.55) |  |
|  | >50 | 35 (18.8) | 228 (10.2) | **2.14 (1.44-3.19)** | **1.67 (1.06-2.63)** | **1.69 (1.07-2.69)** | **1.64 (1.03-2.62)** | 7.5 |
| Poultry (tertiles) | 0 | 55 (29.6) | 794 (35.4) | Ref | Ref | Ref | Ref |  |
|  | >0 to ≤28250 | 64 (34.4) | 689 (30.8) | 1.34 (0.92-1.95) | 1.12 (0.74-1.69) | 1.12 (0.74-1.70) | 1.13 (0.74-1.74) |  |
|  | >28250 | 67 (36.0) | 757 (33.8) | 1.28 (0.88-1.85) | 1.09 (0.70-1.69) | 1.07 (0.69-1.67) | 1.09 (0.69-1.71) |  |

^a^ Model A = adjusted for age and gender

^b^ Model B = model A + adjusted for smoking, education level and BMI

^c^ Model C = model B + adjusted for chronic lung diseases and other comorbidities (based on information extracted from EMR).

^1^ any type of animal farm (main farming category, expressed in tertiles)

^2^ main farm category (any type of animal farm) as registered in the livestock database

^3^ Minimum amount of animals: 50 goats, 250 poultry, 25 pigs, 5 cattle, 5 horses, 50 sheep

^4^ adjusted models (Models A-C) are also corrected for presence of other farms within 500m with a minimum number of animals

^5^ adjusted models (Models A-C) are also corrected for presence of other farms within 1000m with a minimum number of animals

^6^ adjusted models (Models A-C ) are also corrected for presence of other farms within 1500m with a minimum number of animals

^7^ adjusted models (Models A-C ) are also corrected for presence of other farms within 2000m with a minimum number of animals

^8^ adjusted models (Models A-C for goat to sheep) are also corrected for number of other animals within 1000m

^9^ population attributable fraction based on model A
