# Supplementary material for: Livestock-associated risk factors for pneumonia in an area of intensive animal farming in the Netherlands
Source: PLoS One. 2017 Mar 31;12(3):e0174796. doi: 10.1371/journal.pone.0174796 (PMC5376295; doi:10.1371/journal.pone.0174796)
Supplement: S4 Table — (DOCX) [file pone.0174796.s005.docx]

|  |  | Pneumonia cases (%)  n=121 | Non-cases  (%)  n=2305 | Crude OR  (95%CI) | Adjusted OR  Model A^a^  (95%CI) | Adjusted OR  Model B^b^  (95%CI) | Adjusted OR  Model C^c^  (95%CI) |  |
| --- | --- | --- | --- | --- | --- | --- | --- | --- |
| Presence of animal farm with minimum amount of animals within 500m-increments of the residence^1^ | | | | | | | | |
| 500m^2^ | Goat | 9 (7.5) | 33 (1.4) | **5.5 (2.6-11.8)** | **5.1 (2.1-12.4)** | **5.0 (2.0-12.4)** | **4.2 (1.7-10.6)** |  |
|  | Poultry | 26 (21.5) | 328 (14.2) | **1.7 (1.1-2.6)** | 1.3 (0.8-2.3) | 1.3 (0.8-2.3) | 1.3 (0.8-2.3) |  |
|  | Pig | 38 (31.4) | 648 (28.1) | 1.2 (0.8-2.7) | 1.1 (0.7-1.8) | 1.1 (0.7-1.8) | 1.2 (0.7-1.9) |  |
|  | Cattle | 61 (50.4) | 1215 (52.7) | 0.9 (0.6-1.3) | 0.8 (0.5-1.2) | 0.8 (0.5-1.2) | 0.8 (0.5-1.3) |  |
|  | Horse | 32 (26.5) | 539 (23.4) | 1.2 (0.8-1.8) | 0.9 (0.6-1.6) | 0.9 (0.6-1.5) | 0.9 (0.6-1.6) |  |
|  | Sheep | 7 (5.8) | 174 (7.6) | 0.8 (0.3-1.6) | 0.9 (0.4-2.0) | 0.9 (0.4-1.9) | 0.9 (0.4-2.0) |  |
| 1000m^3^ | Goat | 26 (21.5) | 238 (10.3) | **2.4 (1.5-3.7)** | **2.3 (1.4-3.7)** | **2.3 (1.4-3.7)** | **2.2 (1.3-3.6)** |  |
|  | Poultry | 82 (67.8) | 1256 (54.5) | **1.8 (1.2-2.6)** | **1.7 (1.1-2.7)** | **1.7 (1.1-2.7)** | **1.7 (1.1-2.7)** |  |
|  | Pig | 96 (79.3) | 1829 (79.3) | 1.0 (0.6-1.6) | 0.7 (0.4-1.2) | 0.7 (0.4-1.2) | 0.7 (0.4-1.3) |  |
|  | Cattle | 113 (93.4) | 2171 (94.2) | 0.9 (0.4-1.8) | 0.6 (0.3-1.4) | 0.6 (0.3-1.5) | 0.6 (0.3-1.5) |  |
|  | Horse | 95 (78.5) | 1647 (71.5) | 1.5 (0.9-2.3) | 1.4 (0.8-2.4) | 1.4 (0.8-2.4) | 1.4 (0.8-2.4) |  |
|  | Sheep | 35 (28.9) | 724 (31.4) | 0.9 (0.6-1.3) | 0.8 (0.5-1.2) | 0.8 (0.5-1.2) | 0.8 (0.5-1.2) |  |
| 1500m^4^ | Goat | 48 (39.7) | 499 (21.7) | **2.4 (1.6-3.5)** | **2.5 (1.7-3.7)** | **2.5 (1.6-3.6)** | **2.5 (1.7-3.7)** |  |
|  | Poultry | 104 (86.0) | 1942 (84.3) | 1.7 (0.7-1.9) | 0.9 (0.5-1.6) | 0.9 (0.5-1.7) | 0.9 (0.5-1.6) |  |
|  | Pig | 119 (98.4) | 2236 (97.0) | 1.8 (0.4-7.6) | 1.4 (0.3-6.3) | 1.4 (0.3-6.3) | 1.6 (0.3-7.2) |  |
|  | Cattle | 121 (100) | 2298 (99.7) | / | / | / | / |  |
|  | Horse | 112 (92.6) | 2092 (90.8) | 1.3 (0.6-2.5) | 1.2 (0.5-2.7) | 1.2 (0.5-2.8) | 1.3 (0.6-3.1) |  |
|  | Sheep | 65 (53.7) | 1394 (60.5) | 0.8 (0.5-1.1) | 0.7 (0.5-1.1) | 0.8 (0.5-1.1) | 0.7 (0.5-1.1) |  |
| 2000m^5^ | Goat | 65 (53.7) | 767 (33.3) | **2.3 (1.6-3.4)** | **2.3 (1.6-3.4)** | **2.3 (1.5-3.3)** | **2.3 (1.5-3.3)** |  |
|  | Poultry | 117 (96.7) | 2098 (91.0) | **2.9 (1.1-7.9)** | 2.4 (0.8-7.1) | 2.5 (0.9-7.6) | 2.6 (0.9-7.6) |  |
|  | Pig | 121 (100) | 2293 (99.5) | / | / | / | / |  |
|  | Cattle | 121 (100) | 2305 (100) | / | / | / | / |  |
|  | Horse | 118 (97.5) | 2200 (95.4) | 1.9 (0.6-6.0) | 1.2 (0.3-4.0) | 1.1 (0.3-3.9) | 1.2 (0.3-4.1) |  |
|  | Sheep | 94 (77.7) | 1872 (79.3) | 0.9 (0.6-1.4) | 0.9 (0.6-1.4) | 0.9 (0.6-1.4) | 0.9 (0.5-1.4) |  |

^a^Model A = adjusted for age and gender

^b^Model B = model A + adjusted for smoking, education level and BMI

^c^Model C = model B + adjusted for chronic lung diseases and other comorbidities (based on information extracted from EMR).

^1^ Minimum amount of animals: 50 goats, 250 poultry, 25 pigs, 5 cattle, 5 horses, 50 sheep

^2^ adjusted models (Models A-C) are also corrected for presence of other farms within 500m with a minimum number of animals

^3^ adjusted models (Models A-C) are also corrected for presence of other farms within 1000m with a minimum number of animals

^4^ adjusted models (Models A-C ) are also corrected for presence of other farms within 1500m with a minimum number of animals

^5^ adjusted models (Models A-C ) are also corrected for presence of other farms within 2000m with a minimum number of animals
