# Supplementary material for: Livestock-associated risk factors for pneumonia in an area of intensive animal farming in the Netherlands
Source: PLoS One. 2017 Mar 31;12(3):e0174796. doi: 10.1371/journal.pone.0174796 (PMC5376295; doi:10.1371/journal.pone.0174796)
Supplement: S1 File — (PDF) [file pone.0174796.s006.pdf]

VRAGENLIJST

Veehouderij en Gezondheid Omwonenden

Uitleg bij de vragenlijst

Voor u ligt de vragenlijst van het onderzoek ‘Veehouderij en Gezondheid Omwonenden’. Hierin treft u vragen aan over uw beroep, uw gezondheid, contact met dieren, voeding, vrijetijdsbesteding en uw woonomgeving nu en tijdens uw jeugd.

Wij willen u vragen deze vragenlijst vooraf aan uw bezoek aan het onderzoekscentrum in te vullen en mee te nemen naar het onderzoekscentrum. Het invullen van de vragenlijst kost u ongeveer een half uur tijd. Tijdens uw bezoek aan het onderzoekscentrum kunt u eventuele problemen bij het invullen bespreken.

Bij de meeste vragen is het voldoende als u een hokje aankruist. Op een aantal plaatsen in de vragenlijst kunt u uw antwoord toelichten.

Indien u vragen heeft kunt u contact opnemen met:
Floor Borlée; tel. 030- 2532578; email f.borlee@uu.nl
Bij voorkeur tussen 10.00 en 12.00 uur bellen.

A. Algemene vragen

A.1 Op welke dag heeft u deze vragenlijst ingevuld?

|__|__| - |__|__| - |__|__|__|__| (*dd – mm – jjjj*)
dag maand jaar

A.2 Wat is uw geslacht?

❍ Man

❍ Vrouw

A.3 Wat is uw geboortedatum?

|__|__| - |__|__| - |__|__|__|__| (*dd – mm – jjjj*)
dag maand jaar

A.4 Wat is uw geboorteland?

❍ Nederland

❍ Ander land, namelijk ....

A.5 Uit hoeveel personen bestaat uw huishouden, naast u zelf?
(*meerdere antwoorden mogelijk*)

❑ Ik woon alleen

❑ Met partner

❑ Inwonende ouders of andere nog niet genoemde volwassenen: |______| (*aantal*)

❑ Kinderen van 18 jaar of ouder: |______| (*aantal*)

❑ Kinderen 4 t/m 17 jaar: |______| (*aantal*)

❑ Kinderen jonger dan 4 jaar: |______| (*aantal*)

Indien uw huishouden kinderen jonger dan 4 jaar heeft, ...

A.6 Zijn er in het gezin kinderen (jonger dan 4 jaar) die naar een peuterspeelzaal en/of kinderdagverblijf gaan?

❍ ja ❍ nee

B. GEZONDHEID

B.1 Hoe zou u uw gezondheid **op dit moment** omschrijven?

❍ Slecht ❍ Matig ❍ Redelijk ❍ Goed ❍ Uitstekend

B.2 Bent u de **afgelopen 12 maanden** in een Nederlands ziekenhuis opgenomen geweest?

❍ Nee

❍ Ja, vanwege ....

B.3 Bent u de **afgelopen 12 maanden** in een buitenlands ziekenhuis opgenomen geweest?

❍ Nee

❍ Ja, vanwege ....

In welk land? ....

B.4 Heeft u ooit astma gehad?

❍ Ja ❍ Nee (***zo nee**, ga door met vraag B.10*)

Zo ja, ...

B.5 Werd de astma door een arts bevestigd?

❍ Nee

❍ Ja, dat was in het jaar |__|__|__|__| (*jaartal*)

B.6 Hoe oud was u toen u uw eerste astma-aanval had?

Ik was toen |______| jaar oud

B.7 Heeft u in **de afgelopen 12 maanden** een astma-aanval gehad?

❍ Ja ❍ Nee (***zo nee**, ga door met vraag B.9*)

Zo ja, ...

B.8 Hoeveel astma aanvallen heeft u in **de afgelopen 12 maanden** gehad?

|______| aanvallen

B.9 Gebruikt u op dit moment geneesmiddelen tegen astma? (*bijv. inhalatoren, aerosols, tabletten*)

❍ Ja ❍ Nee

B.10 Selecteer per rij het vakje dat het beste beschrijft hoe u zich de afgelopen week heeft gevoeld.

Hoe vaak voelde u zich in de afgelopen week ……	Nooit	Zelden	Af en Toe	Regel-matig	Heel vaak	Meestal	Altijd
1. Kortademig in rust?	❍	❍	❍	❍	❍	❍	❍
2. Kortademig gedurende lichamelijke inspanning?	❍	❍	❍	❍	❍	❍	❍
3. Angstig / bezorgd voor de volgende benauwd-heidsaanval?	❍	❍	❍	❍	❍	❍	❍
4. Neerslachtig vanwege uw ademhalingsproblemen?	❍	❍	❍	❍	❍	❍	❍

Hoe vaak in de afgelopen week heeft u ……	Nooit	Zelden	Af en Toe	Regel-matig	Heel vaak	Meestal	Altijd
5. **Gehoest**?	❍	❍	❍	❍	❍	❍	❍
6. **Slijm** opgehoest?	❍	❍	❍	❍	❍	❍	❍

In welke mate voelde u zich in de afgelopen week beperkt door ademhalingsproblemen bij het uitvoeren van ……	Helemaal niet beperkt	Héél weinig beperkt	Een beetje beperkt	Tamelijk beperkt	Erg beperkt	Héél erg beperkt	Volledig beperkt / niet mogelijk
7. Zware lichamelijke activiteiten (trap lopen, haasten, sporten)	❍	❍	❍	❍	❍	❍	❍
8. Matige lichamelijke activiteiten (wandelen, huishoudelijk werk, boodschappen doen)	❍	❍	❍	❍	❍	❍	❍
9. Dagelijkse activiteiten (u zelf aankleden, wassen)	❍	❍	❍	❍	❍	❍	❍
10. Sociale activiteiten (praten, omgaan met kinderen, vrienden / familie bezoeken)	❍	❍	❍	❍	❍	❍	❍

B.11 Bent u ooit ergens overgevoelig of allergisch voor (geweest)?

❍ Ja ❍ Nee (***zo nee**, ga door met vraag B.14*)

Zo ja, ...

B.12 Wilt u aankruisen voor welke stoffen u overgevoelig of allergisch bent?
En zo ja, kunt u dan aangeven wat de aard van de klachten is die u daarbij heeft?
(*meerdere antwoorden mogelijk*)

Overgevoelig of allergisch voor?		***Zo ja**, klachten bij overgevoeligheid of allergie …* Niezen of loopneus	Benauwdheid	Jeukende of rode huid	Jeukende of tranende ogen
**A.** Huisstof	❍ Nee ❍ Ja →	❑	❑	❑	❑
**B.** Voedsel	❍ Nee ❍ Ja →	❑	❑	❑	❑
**C.** Dieren	❍ Nee ❍ Ja →	❑	❑	❑	❑
**D.** Planten of pollen	❍ Nee ❍ Ja →	❑	❑	❑	❑
❑ Anders, namelijk		❑	❑	❑	❑

***Als u overgevoelig of allergisch bent voor dieren (C)**, ..*

B.13 Voor welke dieren bent u allergisch?
(*meerdere antwoorden mogelijk*)

❑ Kat ❑ Hond ❑ Konijn, cavia of hamster

❑ Paard ❑ Vogel

❑ Overig, namelijk ....

B.14 Heeft u ooit eczeem of een vorm van huidallergie gehad?

❍ Ja ❍ Nee

B.15 Heeft u in **de afgelopen 3 jaar** een longontsteking gehad?

❍ Ja, 1 keer ❍ Ja, 2 keer ❍ Ja, 3 keer of vaker

❍ Nee (***zo nee**, ga door met vraag B.19*)

Zo ja, ...

B.16 In welk jaar heeft u in **de afgelopen 3 jaar** een longontsteking gehad?
Als u de afgelopen 3 jaar meer dan één longontsteking heeft gehad, moet u voor deze en de volgende vragen de keer in gedachten nemen dat u er het ergst aan toe was.

|__|__|__|__| (jaartal)

B.17 Kunt u aangeven wat van toepassing was toen u een longontsteking had? (*meerdere antwoorden mogelijk*)

❑ De huisarts vertelde mij dat ik een longontsteking had

❑ De huisarts schreef mij antibiotica voor

❑ De huisarts verwees mij naar de specialist

❑ De specialist vertelde mij dat ik een longontsteking had

❑ Er is een longfoto van mij gemaakt

❑ De huisarts/specialist vertelde dat op de longfoto een longontsteking zichtbaar was

❑ De specialist schreef mij antibiotica voor

❑ Ik werd in het ziekenhuis opgenomen waar ik |______| dagen bleef

B.18 Wat ik mij **vooral** herinner van toen de longontsteking begon is:
(*meerdere antwoorden mogelijk*)

❑ Koorts ❑ Benauwdheid ❑ Hoesten

❑ Spierpijn ❑ Pijn op mijn borst ❑ Piepende ademhaling

❑ Maagpijn ❑ Vermoeidheid ❑ Gevoel van somberheid

B.19 Neemt u deel aan de jaarlijkse griepvaccinatie (griepprik)?

❍ Ja ❍ Nee (***zo nee**, ga door met vraag B.21*)

Zo ja, ...

B.20 Sinds wanneer heeft u deelgenomen aan deze jaarlijkse griepvaccinatie?

❍ Ik heb afgelopen jaar voor het eerst deelgenomen

❍ Sinds 2 tot 5 jaar

❍ Sinds meer dan 5 jaar

B.21 Hieronder ziet u een lijst met verschillende gezondheidsklachten. We vragen u om per gezondheidsklacht in kolom **A** aan te kruisen of u hier de afgelopen maand last van heeft gehad. Indien 'Ja': ga door met kolom **B**.

A. *Afgelopen maand last gehad van deze klacht?*		B. *Heeft u voor deze klacht (het afgelopen jaar) een huisarts bezocht?* Nee	Ja, in de afgelopen 4 weken	Ja, tussen 1 en 12 maanden geleden
1. moeheid	❑ Ja →	❍	❍	❍
2. buik- of maagklachten	❑ Ja →	❍	❍	❍
3. misselijkheid	❑ Ja →	❍	❍	❍
4. diarree	❑ Ja →	❍	❍	❍
5. verstopping	❑ Ja →	❍	❍	❍
6. bloed en/of slijm in de ontlasting	❑ Ja →	❍	❍	❍
7. braken	❑ Ja →	❍	❍	❍
8. koorts (38 °C of hoger)	❑ Ja →	❍	❍	❍
9. irritatie aan het oog	❑ Ja →	❍	❍	❍
10. oorklachten	❑ Ja →	❍	❍	❍
11. hartkloppingen	❑ Ja →	❍	❍	❍
12. nek- of schouderklachten	❑ Ja →	❍	❍	❍
13. rugklachten	❑ Ja →	❍	❍	❍
14. pijn of druk op de borst	❑ Ja →	❍	❍	❍
15. klachten aan arm / elleboog / hand/pols	❑ Ja →	❍	❍	❍
16. klachten aan been / heup / knie / voet	❑ Ja →	❍	❍	❍
17. spierpijn	❑ Ja →	❍	❍	❍
18. hoofdpijn	❑ Ja →	❍	❍	❍
19. duizeligheid of licht in het hoofd	❑ Ja →	❍	❍	❍
20. angstig / nerveus / gespannen gevoel	❑ Ja →	❍	❍	❍
21. depressief gevoel	❑ Ja →	❍	❍	❍
22. plotselinge (hevige) stress of crisis	❑ Ja →	❍	❍	❍
23. prikkelbaar / boosheid	❑ Ja →	❍	❍	❍
24. slaapproblemen	❑ Ja →	❍	❍	❍
25. toegenomen gebruik van drank, sigaretten, drugs, slaap- of kalmeringsmiddelen	❑ Ja →	❍	❍	❍
26. benauwd of kortademig in rust (zonder inspanning)	❑ Ja →	❍	❍	❍
27. keelpijn	❑ Ja →	❍	❍	❍
28. hoesten	❑ Ja →	❍	❍	❍
29. klachten aan de neus (bv. vaak niezen, prikkelend gevoel of regelmatig verstopte neus)	❑ Ja →	❍	❍	❍
30. huidproblemen (bv. jeuk, uitslag of rode plekken)	❑ Ja →	❍	❍	❍
31. plasproblemen	❑ Ja →	❍	❍	❍
32. verandering in gewicht	❑ Ja →	❍	❍	❍
❑ andere klachten, namelijk		❍	❍	❍

❑ Ik had de afgelopen maand **geen** last van bovengenoemde gezondheidsklachten (***ga door*** *met deel C*)

U heeft hiernaast gezondheidsklachten met ja aangekruist, ...

B.22 Houden, volgens u, de gezondheidsklachten die u heeft aangekruist mogelijk verband met de aanwezigheid van veehouderij(en) in uw woonomgeving?

❍ Ja ❍ Nee (***zo nee***, *ga door met deel C*)

Zo ja, ...

B.23 Door welke factor(en) worden deze gezondheidsklachten mogelijk veroorzaakt?
(*meerdere antwoorden mogelijk*)

❑ Fijnstof ❑ Geur

❑ Geluid ❑ Overdraagbare dierziekten (zoals bijv. MRSA of Q-koorts)

❑ Overige, namelijk ....

....

C. Werk en opleiding

C.1 Wat is de **hoogste** opleiding die u heeft **afgemaakt**?

- ❍ Geen opleiding afgemaakt
- ❍ Lager onderwijs (basisschool)
- ❍ Lager / voorbereidend beroepsonderwijs (LTS, LEAO, LHNO, VMBO)
- ❍ Middelbaar voortgezet onderwijs (MAVO, MULO, MBO-kort, VMBO-t)
- ❍ Middelbaar beroepsonderwijs (MBO-lang, MTS, MEAO, BOL, BBS, INAS)
- ❍ Middelbaar hoger onderwijs (HAVO, VWO, Atheneum, Gymnasium, HBS, MMS)
- ❍ Hoger beroepsonderwijs (HBO, HTS, HEAO)
- ❍ Universiteit

C.2 Wat zijn uw **voornaamste** werkzaamheden?
(*meerdere antwoorden mogelijk*)

- ❑ Ik werk in dienstverband of als zelfstandige (minder dan 19 uur per week)
- ❑ Ik werk in dienstverband of als zelfstandige (19 uur per week of meer)
- ❑ Ik verzorg het eigen huishouden
- ❑ Ik ben werkloos / werkzoekend
- ❑ Ik volg onderwijs / studeer
- ❑ Ik ben arbeidsongeschikt, WAO, AAW
- ❑ Ik ben gepensioneerd, in de VUT
- ❑ Ik doe vrijwilligerswerk

C.3 Wat is uw werkadres (of, als u studeert, het adres van uw onderwijsinstelling)?

- ❍ Niet van toepassing (***ga door** met vraag C.5*)
- ❍ Ik werk op meerdere locaties (*vul de plaats in waar u het **vaakst werkt***)
- ❍ Ik werk op de volgende locatie

S.v.p. geen postbusadres invullen, ...

STRAAT: .... HUISNUMMER: ....

PLAATS: .... POSTCODE: ....

C.4 Hoeveel uur **per week** gebruikt u gewoonlijk onderstaande vervoermiddelen voor uw **reis** van huis naar werk/school en terug?

Vervoermiddel	*In de herfst / winter*	*In de lente / zomer*
**1.** Trein en bus	____ *(uur / week)*	____ *(uur / week)*
**2.** Auto	____ *(uur / week)*	____ *(uur / week)*
**3.** Brommer, scooter, motor	____ *(uur / week)*	____ *(uur / week)*
**4.** Fiets	____ *(uur / week)*	____ *(uur / week)*
**5.** Te voet	____ *(uur / week)*	____ *(uur / week)*

C.5 Met welke van de volgende groepen heeft u in uw (vrijwilligers)werk of opleiding **persoonlijk** contact (dus niet alleen via telefoon of e-mail)?

❑ Patiënten ❑ Bewoners verpleeg- of verzorgingshuis

❑ Klanten ❑ Kinderen/leerlingen (0-12 jaar) ❑ Dieren

❑ Niet van toepassing

C.6 Heeft u ooit een **functie / beroep** uitgeoefend waarbij u werd **blootgesteld** aan dampen, gassen, stof of rook?

❍ Ja ❍ Nee

C.7 Wilt u hieronder alle functies / beroepen (maximaal 6) opschrijven die u ooit heeft uitgeoefend?
(*Begin met uw huidige of laatste beroep en eindig met uw eerste beroep. Als u de jaartallen niet meer weet kunt u ook het aantal jaren in deze functie opschrijven*)

Bijvoorbeeld:

a. *soort functie / beroep* — *taken / activiteiten*

. *operator* .... *bedienen verpakkingslijn* ....

soort bedrijf / instelling / industrietak — *van* (jaar) — *tot* (jaar) — (aantal jaar)

. *zaaizaadbedrijf* .... 2 0 0 1 | 2 0 1 4 | ____

(huidige/laatste beroep)

1. *soort functie / beroep* — *taken / activiteiten*

....

....

soort bedrijf / instelling / industrietak — *van* (jaar) — *tot* (jaar) — (aantal jaar)

.... |__|__|__|__| |__|__|__|__| |____|

....

2. *soort functie / beroep* — *taken / activiteiten*

....

....

soort bedrijf / instelling / industrietak — *van* (jaar) — *tot* (jaar) — (aantal jaar)

.... |__|__|__|__| |__|__|__|__| |____|

....

3. *soort functie / beroep* — *taken / activiteiten*

....

....

soort bedrijf / instelling / industrietak — *van* (jaar) — *tot* (jaar) — (aantal jaar)

.... |__|__|__|__| |__|__|__|__| |____|

....

(*vervolg*)

Bijvoorbeeld:

b. *soort functie / beroep* — *taken / activiteiten*

. *heftruckchauffeur* .... *aanvoer/afvoer producten in magazijn* ....

soort bedrijf / instelling / industrietak — *van* (jaar) — *tot* (jaar) — (aantal jaar)

. *diervoederbedrijf* .... 1 9 8 3 — 2 0 0 0 — ___

---

4. *soort functie / beroep* — *taken / activiteiten*

....

....

soort bedrijf / instelling / industrietak — *van* (jaar) — *tot* (jaar) — (aantal jaar)

.... |__|__|__|__| |__|__|__|__| |____|

....

---

5. *soort functie / beroep* — *taken / activiteiten*

....

....

soort bedrijf / instelling / industrietak — *van* (jaar) — *tot* (jaar) — (aantal jaar)

.... |__|__|__|__| |__|__|__|__| |____|

....

(eerste beroep)

---

6. *soort functie / beroep* — *taken / activiteiten*

....

....

soort bedrijf / instelling / industrietak — *van* (jaar) — *tot* (jaar) — (aantal jaar)

.... |__|__|__|__| |__|__|__|__| |____|

....

D. Wonen

D.1 In welke gemeente/woonplaats heeft u het grootste deel van uw jeugd (tot 18 jaar) doorgebracht?

....

D.2 Heeft u als kind (tot 18 jaar) op een boerderij met dieren gewoond?

❍ Nee (***zo nee**, ga door met vraag D.4*)

❍ Ja, namelijk van leeftijd |______| (*jaar oud*)

tot leeftijd |______| (*jaar oud*)

Zo ja, ...

D.3 Welke van de onderstaande boerderijtypes is van toepassing voor de boerderij waar u als kind (tot 18 jaar) heeft gewoond?
(*meerdere antwoorden mogelijk*)

❑ Akkerbouw of tuinbouw

❑ Melkrundveehouderij

❑ Vleeskalverhouderij

❑ Geitenhouderij

❑ Schapenhouderij

❑ Varkenshouderij (fokzeugen, vleesvarkens, etc.)

❑ Pluimveehouderij (legkippen, vleeskuikens, kalkoenen, etc.)

❑ Manege, paardenfokkerij

❑ Anders, namelijk ....

D.4 Verbleef u als kind (tot 18 jaar) tijdens vakanties wel eens op een boerderij met dieren?

❍ Ja ❍ Nee

D.5 Heeft u als kind (tot 18 jaar) één of meerdere van onderstaande werkzaamheden uitgevoerd op een boerderij?
(*meerdere antwoorden mogelijk*)

❑ Dierverzorging met intensief diercontact

❑ Werken met mest

❑ Werken met stro/hooi/kuilgras/diervoeder

❑ Gewasverzorging

❑ Geen van bovenstaande antwoorden

D.6 Sinds wanneer woont u in uw huidige woning?

|__|__| - |__|__|__|__| (*mm – jjjj*)
maand jaar

D.7 Mijn woning is een:

❍ huurwoning ❍ koopwoning

D.8 Bent u van plan om binnen twee jaar te verhuizen?

❍ Nee (***zo nee**, ga door met vraag D.10*)

❍ Wel van plan ❍ Eventueel wel / misschien

Indien u (eventueel) van plan bent binnen twee jaar te verhuizen, ...

D.9 Wat is de reden van uw verhuiswens?
(*meerdere antwoorden mogelijk*)

❑ Gezondheid ❑ Studie of werk

❑ De woning (bijv. een grotere of kleinere woning) ❑ De woonomgeving

❑ Geen van bovenstaande antwoorden

D.10 Hoeveel uur per etmaal (24 uur) brengt u door in of om uw eigen huis (inclusief slapen) op een gemiddelde doordeweekse dag?

❍ Minder dan 8 uur per dag ❍ 8 - 15 uur per dag

❍ 16 - 19 uur per dag ❍ 20 - 24 uur per dag

D.11 Heeft u **afgelopen 12 maanden** last gehad van schimmelplekken in uw woning?
(*bijv. op plafond, muur of vloer*)

❍ Ja ❍ Nee

D.12 Heeft u **afgelopen 12 maanden** waterschade of lekkage in uw woning gehad?

❍ Ja ❍ Nee

D.13 Heeft u **afgelopen 12 maanden** last gehad van vochtplekken in uw woning?

❍ Ja ❍ Nee

D.14 Heeft u **de afgelopen 5 jaar** huisdieren gehouden?

❍ Ja ❍ Nee (*zo nee, ga door met vraag D.16*)

Zo ja, ...

D.15 Welke soort huisdier(en) heeft u **de afgelopen 5 jaar** gehouden?
(*voor elk van de onderstaande huisdieren geeft u aan wat van toepassing is*)

Huisdier ...	*Nee, ook niet in de afgelopen 5 jaar*	*Ja, ik heb dit huisdier nu*	*Ja, niet nu, maar in de afgelopen 5 jaar wel gehad*
**1.** Kat	❍	❍	❍
**2.** Hond	❍	❍	❍
**3.** Vogel	❍	❍	❍
**4.** Konijn, cavia of hamster	❍	❍	❍
**5.** Muis of rat	❍	❍	❍
**6.** Vis	❍	❍	❍
**7.** Schildpad	❍	❍	❍

D.16 Heeft u **de afgelopen 5 jaar** landbouwhuisdieren gehouden als hobby?

❍ Ja ❍ Nee (*zo nee, ga door met vraag D.18*)

Zo ja, ...

D.17 Welke soort landbouwhuisdier(en) heeft u **de afgelopen 5 jaar** als hobby gehouden?
(*voor elk van de onderstaande landbouwhuisdieren geeft u aan wat van toepassing is*)

Landbouwhuisdier ...	*Nee, ook niet in de afgelopen 5 jaar*	*Ja, ik heb dit huisdier nu*	*Ja, niet nu, maar in de afgelopen 5 jaar wel gehad*
**1.** Varken	❍	❍	❍
**2.** Koe	❍	❍	❍
**3.** Schaap	❍	❍	❍
**4.** Geit	❍	❍	❍
**5.** Kip, kalkoen, eend of gans	❍	❍	❍
**6.** Paard, pony of ezel	❍	❍	❍

D.18 Hoe groot schat u de afstand tussen uw woning en de **dichtstbijzijnde veehouderij**?

❍ Minder dan 500 meter

❍ 500 - 1000 meter

❍ Meer dan 1000 meter

E. Roken

E.1 Heeft u (ooit) sigaretten, sigaren en/of pijpen gerookt?
(*'Ja' betekent minimaal 20 pakjes sigaretten in totaal, of minimaal 1 jaar lang 1 sigaret per dag*)

- ❍ Nee (***zo nee***, *ga door met vraag E.4*)
- ❍ Ja, ik heb vroeger gerookt, maar ik ben ⌊______⌋ jaar geleden gestopt
- ❍ Ja, ik rook (nog steeds)

Zo ja, ...

E.2 Hoe oud was u toen u begon met roken?

Ik was toen ⌊______⌋ jaar oud

E.3 Hoeveel sigaretten, sigaren of pijpen rookt of rookte u **gemiddeld per dag**?
(*1 pakje shag = 40 sigaretten; meerdere antwoorden mogelijk*)

a. Sigaretten: ⌊______⌋ per dag

b. Sigaren: ⌊______⌋ per dag

c. Pijp: ⌊______⌋ per dag

E.4 Wordt er bij u thuis **binnenshuis** gerookt?

❍ Zelden of nooit ❍ Soms ❍ Regelmatig ❍ Vrijwel dagelijks

E.5 Rookt men in de ruimte waarin u werkt?

❍ Zelden of nooit ❍ Soms ❍ Regelmatig ❍ Vrijwel dagelijks

E.6 Bent u in **de afgelopen 12 maanden** blootgesteld geweest aan tabaksrook van anderen?

❍ Zelden of nooit (***ga door*** *met deel F*)

❍ Soms ❍ Regelmatig ❍ Vrijwel dagelijks

Indien van toepassing, ...

E.7 Hoeveel uur wordt u gemiddeld per dag blootgesteld aan de tabaksrook van anderen?
(*graag afronden op hele uren; bij minder dan een half uur kunt u 0 invullen*)

Ongeveer ⌊______⌋ uur per dag

F. Voeding

F.1 Volgt u een specifiek voedingspatroon t.a.v. vlees en/of vis?
(*meerdere antwoorden mogelijk*)

- ❑ Ja, ik eet geen vlees meer sinds |______| aantal jaar
- ❑ Ja, ik eet geen vis meer sinds |______| aantal jaar
- ❑ Ja, eet geen dierlijke producten meer (ook geen zuivel, eieren) sinds |______| aantal jaar
- ❑ Ja, ik eet koosjer of halal sinds |______| aantal jaar
- ❑ Nee, ik heb geen specifiek voedingspatroon t.a.v. vlees en vis

F.2 Heeft u in **de afgelopen maand** één van de onderstaande producten gegeten?
(*meerdere antwoorden mogelijk*)

- ❑ Rauw rundvlees (bijvoorbeeld filet americain, ossenworst, carpaccio, tartaar, biefstuk)
- ❑ Varkensvlees
- ❑ Gevogelte (bijvoorbeeld kipfilet)
- ❑ Rauwe vis (bijvoorbeeld haring, gerookte zalm, sushi)
- ❑ Rauwe, ongekookte melk van de boerderij
- ❑ Rauwmelkse zachte kaas (bijvoorbeeld roquefort, gorgonzola)
- ❑ Rauwe eieren (bijvoorbeeld in zelfbereide toetjes of zelfgemaakte mayonaise)
- ❑ Krop sla of voorgesneden sla
- ❑ Kiemgroenten (taugé, alfalfa)
- ❑ Nee, ik heb geen van deze producten gegeten

F.3 Hoe vaak eet u **gewoonlijk** de volgende voedingsmiddelen?

Hoe vaak eet u ...	Meer dan 3 keer per week	1 tot 3 keer per week	1 tot 3 keer per maand	Een paar keer per jaar	Nooit
1. Rauw rundvlees	❍	❍	❍	❍	❍
2. Varkensvlees	❍	❍	❍	❍	❍
3. Gevogelte	❍	❍	❍	❍	❍
4. Rauwe vis	❍	❍	❍	❍	❍
5. Rauwe melk van de boerderij	❍	❍	❍	❍	❍
6. Rauwmelkse zachte kaas	❍	❍	❍	❍	❍
7. Rauwe eieren	❍	❍	❍	❍	❍
8. Krop sla of voorgesneden sla	❍	❍	❍	❍	❍
9. Kiemgroenten (taugé, alfalfa)	❍	❍	❍	❍	❍

G. Vrijetijdsbesteding

G.1 Bent u in **de afgelopen 12 maanden** in het buitenland geweest?

❍ Ja ❍ Nee (*zo* ***nee***, *ga door met vraag G.3*)

Zo ja, ...

G.2 Naar welke landen bent u geweest en hoe lang verbleef u daar?
(*bij meer dan 5 reizen, vult u de 5 langst durende reizen in*)

Naar welke landen?	*Hoe lang verbleef u daar?*
**1.**	_____ aantal dagen
**2.**	_____ aantal dagen
**3.**	_____ aantal dagen
**4.**	_____ aantal dagen
**5.**	_____ aantal dagen

G.3 Bent u **de afgelopen 12 maanden** wel eens in de tuin of op het land bezig geweest, waarbij u met uw blote handen in de aarde werkte?

❍ Ja ❍ Nee (*zo* ***nee***, *ga door met vraag G.5*)

Zo ja, ...

G.4 Hoeveel uur per week (in de lente/zomer) heeft u hieraan gemiddeld besteed?
(*graag afronden op hele uren; bij minder dan een half uur kunt u 0 invullen*)

Gemiddeld _____ uur aan besteed

G.5 Heeft u in de afgelopen 12 maanden mest gebruikt in de tuin?
(*meerdere antwoorden mogelijk*)

❑ Ja, dierlijke mest ❑ Ja, kunstmest ❑ Ja, compost

❑ Nee

G.6 Heeft u in **de afgelopen 12 maanden** een boerderij bezocht?
(*meerdere antwoorden mogelijk*)

- ❑ Ja, vanwege bezoek aan familie, vrienden of kennissen
- ❑ Ja, vanwege mijn werk
- ❑ Ja, om producten te kopen (bijv. groente, fruit, eieren of vlees)
- ❑ Ja, een kinderboerderij
- ❑ Ja, om een andere reden
- ❑ Nee (***zo nee***, *ga door met vraag G.8*)

Zo ja, ...

G.7 Met welke diersoort heeft u tijdens het (kinder)boerderijbezoek contact gehad?
(dat wil zeggen dat u het dier zelf of de uitwerpselen van het dier heeft aangeraakt)
(*meerdere antwoorden mogelijk*)

- ❑ Varken
- ❑ Koe
- ❑ Schaap
- ❑ Geit
- ❑ Kip, kalkoen, eend of gans
- ❑ Paard, pony of ezel
- ❑ Konijn, cavia of hamster
- ❑ Ik heb geen contact met bovenstaande diersoorten gehad

G.8 Neem een gewone week in uw gedachten. Hoeveel uur heeft u toen buiten doorgebracht in uw vrije tijd met de volgende activiteiten?
(*graag afronden op hele uren; bij minder dan een half uur kunt u 0 invullen*)

Activiteit buiten in vrije tijd	*In de herfst / winter*	*In de lente / zomer*
**1.** Lopen (bijvoorbeeld van en naar winkels, wandelingen, hond uitlaten)	____ *(uur / week)*	____ *(uur / week)*
**2.** Fietsen (bijvoorbeeld van en naar winkels, fietstocht maken)	____ *(uur / week)*	____ *(uur / week)*
**3.** Buitensporten (bijvoorbeeld joggen, tennissen, golf, enz.)	____ *(uur / week)*	____ *(uur / week)*
**4.** Om het huis bezig zijn (tuinieren, dieren verzorgen, doe-het-zelven, in de tuin zitten)	____ *(uur / week)*	____ *(uur / week)*
**5.** Overige buitenactiviteiten	____ *(uur / week)*	____ *(uur / week)*

H. Woonomgeving

H.1 Hieronder leggen wij u een aantal stellingen voor over wat u vindt en merkt van de veehouderij vlak bij uw huis.
Het gaat daarbij om de veehouderij in het algemeen, en niet alleen om de grotere bedrijven

In hoeverre met u het eens/oneens ...	Zeer oneens	Oneens	Neutraal	Eens	Zeer eens
1. In mijn woonomgeving staan veel veehouderijen	❍	❍	❍	❍	❍
2. De veehouderij belast mijn woonomgeving zwaar	❍	❍	❍	❍	❍
3. De boeren doen hun best om ernstige overlast in mijn woonomgeving te voorkomen	❍	❍	❍	❍	❍
4. De veehouderijen zijn van groot belang voor de Nederlandse economie	❍	❍	❍	❍	❍
5. Ik ben blij met veehouders in mijn woonomgeving	❍	❍	❍	❍	❍
6. Er wordt teveel gediscussieerd over de nadelen van de veehouderij	❍	❍	❍	❍	❍
7. De geur van het uitrijden van mest stoort mij iedere keer weer	❍	❍	❍	❍	❍
8. De veehouderij bedreigt mijn gezondheid	❍	❍	❍	❍	❍
9. Ik heb het idee dat de bedreiging van de veehouderij voor mijn gezondheid groter is dan 10 jaar geleden	❍	❍	❍	❍	❍
10. De veehouderij vormt geen risico voor mijn gezondheid als de veehouders de diergezondheid goed bewaken	❍	❍	❍	❍	❍
11. Ik maak me ongerust over de invloed van het gebruik van antibiotica in de veehouderij op mijn gezondheid	❍	❍	❍	❍	❍
12. Ik ben ongerust over nieuwe ziekten die van dieren over kunnen gaan op mensen	❍	❍	❍	❍	❍
13. Ik heb gezondheidsklachten die veroorzaakt worden door veehouderij in mijn omgeving	❍	❍	❍	❍	❍
14. Een veehouder houdt van zijn dieren en zorgt er goed voor	❍	❍	❍	❍	❍
15. Zolang ik voor mijzelf of mijn gezin geen overlast ervaar, mag de veehouderij doorgroeien	❍	❍	❍	❍	❍
16. Door de bouw van grotere stallen wordt het landschap verstoord	❍	❍	❍	❍	❍
17. Ik eet zelf minder vlees dan 10 jaar geleden	❍	❍	❍	❍	❍

H.2 Hoe vaak heeft u in **de afgelopen 12 maanden** bij u thuis de geur geroken van onderstaande bronnen?
Het gaat daarbij om de veehouderij in het algemeen, en niet alleen om de grotere bedrijven

Bron ...	Dagelijks	Minstens 1× per week	Minstens 1× per maand	Minstens 1× in afgelopen jaar	Niet in afgelopen jaar (nooit)
1. Mengvoederbedrijf	❍	❍	❍	❍	❍
2. Landbouw / veehouderij algemeen	❍	❍	❍	❍	❍
3. Varkenshouderij	❍	❍	❍	❍	❍
4. Pluimveehouderij	❍	❍	❍	❍	❍
5. Rundveehouderij	❍	❍	❍	❍	❍
6. Mest uitrijden	❍	❍	❍	❍	❍
7. Open haard, allesbrander of vuurkorf van buren	❍	❍	❍	❍	❍
8. Overige, namelijk:	❍	❍	❍	❍	❍

H.3 Als u denkt aan **de afgelopen 12 maanden**, welk getal van 0 t/m 10 geeft het beste aan in welke mate u thuis gehinderd wordt door geur van onderstaande bronnen?

		Helemaal niet gehinderd		←					→		*Extreem gehinderd*	
Bron ...	Niet ruikbaar	0	1	2	3	4	5	6	7	8	9	10
1. Mengvoederbedrijf	❍	❍	❍	❍	❍	❍	❍	❍	❍	❍	❍	❍
2. Landbouw / veehouderij algemeen	❍	❍	❍	❍	❍	❍	❍	❍	❍	❍	❍	❍
3. Varkenshouderij	❍	❍	❍	❍	❍	❍	❍	❍	❍	❍	❍	❍
4. Pluimveehouderij	❍	❍	❍	❍	❍	❍	❍	❍	❍	❍	❍	❍
5. Rundveehouderij	❍	❍	❍	❍	❍	❍	❍	❍	❍	❍	❍	❍
6. Mest uitrijden	❍	❍	❍	❍	❍	❍	❍	❍	❍	❍	❍	❍
7. Open haard, allesbrander of vuurkorf van buren	❍	❍	❍	❍	❍	❍	❍	❍	❍	❍	❍	❍
8. Overige, namelijk:	❍	❍	❍	❍	❍	❍	❍	❍	❍	❍	❍	❍

H.4 Geef voor onderstaande uitspraken aan hoe vaak deze, als gevolg van geur van veehouderij(en) in uw woonomgeving, op u van toepassing zijn:

In hoeverre van toepassing ...	Nooit	Soms	Regelmatig	Vaak
1. Thuiskomen wordt minder plezierig	❍	❍	❍	❍
2. Het stoort tijdens gesprekken	❍	❍	❍	❍
3. Ik slaap onrustig	❍	❍	❍	❍
4. Ik zoek een oplossing voor de overlast	❍	❍	❍	❍
5. Ik nodig minder vaak vrienden thuis uit	❍	❍	❍	❍
6. Ik vind het niet fijn om thuis te zijn	❍	❍	❍	❍
7. Er zijn spanningen binnen het gezin	❍	❍	❍	❍
8. Ik klaag bij de veehouder	❍	❍	❍	❍
9. Ik heb minder zin om naar buiten te gaan	❍	❍	❍	❍
10. Ik doe de ramen dicht	❍	❍	❍	❍
11. Ik hang de was niet buiten	❍	❍	❍	❍
12. Ik klaag bij de gemeente	❍	❍	❍	❍
13. Ik probeer aan wat anders te denken en zoek afleiding	❍	❍	❍	❍
14. Ik kom niet in slaap	❍	❍	❍	❍

H.5 Denkt u dat de geur van veehouderij(en) schadelijk is voor de gezondheid?

❍ Nee

❍ Ja

❍ Geen mening

Opmerkingen

……………………………………………………………………………………………

……………………………………………………………………………………………

……………………………………………………………………………………………

……………………………………………………………………………………………

HARTELIJK DANK VOOR HET INVULLEN VAN DE VRAGENLIJST!!!

Vragenlijst voor Veehouderij en Gezondheid Omwonenden 2014
